# Breaking Stiffness‐Tunability Trade‐offs in Metamaterials: a Minimal Surface Guided Hybrid Lattice Strategy

**DOI:** 10.1002/advs.202510586

**Published:** 2025-08-11

**Authors:** Min Zhang, Kang Gao, Jinlong Liu, Zhiqiang Zou, Jie Yang, Ma Qian, Wei Zhai, Zhangming Wu

**Affiliations:** ^1^ Key Laboratory of Concrete and Prestressed Concrete Structures of the Ministry of Education School of Civil Engineering Southeast University Nanjing 211189 China; ^2^ School of Engineering Cardiff University The Parade Cardiff CF24 3AA UK; ^3^ School of Engineering RMIT University Melbourne VIC 3000 Australia; ^4^ Department of Mechanical Engineering National University of Singapore Singapore 117575 Singapore

**Keywords:** additive manufacturing, finite element model, homogenization theory, hybrid lattice metamaterials, mechanical property, triply periodic minimal surfaces

## Abstract

A longstanding trade‐off between stiffness and tunability has significantly constrained the multifunctional potential of architected metamaterials. Here, a generalizable design framework is introduced that integrates shell‐ and plate‐based lattice architectures via a spatially compensated Boolean fusion strategy. The design enables tunable architectures with optimized mechanical robustness. The capability is demonstrated through two representative configurations: one based on Primitive TPMS and one on IWP TPMS, each fused with simple cubic plate lattices. The resulting structures are fabricated with high geometric fidelity using PolyJet printing and evaluated across multiple scales using homogenization, quasi‐static compression testing, and finite element analysis. Compared with similarly ultrastiff plate lattices, the hybrid structure achieves a 213.98% increase in the tunable range of effective elastic modulus. The hybrid lattices reach 137.34% and 110.84% of the Hashin‐Shtrikman upper bound for Young's modulus at relative densities of 0.33 and 0.34, respectively. Compared to single lattices, the hybrid designs show significant improvements: ultimate stress increased by up to 690% and specific energy absorption increased by 110%. The proposed metamaterials offer excellent tunability and mechanical performance, providing the flexibility to tailor structural behaviors for diverse applications such as biomedical engineering, acoustic isolation, and intelligent infrastructure systems.

## Graphical abstract

This work breaks the persistent stiffness‐tunability compromise in metamaterials through a spatial Boolean fusion strategy integrating Primitive/IWP TPMS shells with cubic plate lattices. The framework enables highly tunable architectures with optimized robustness, fabricated via PolyJet printing. Multi‐scale validation through homogenization, quasi‐static compression, and FEA confirms exceptional mechanical performance: achieving stiffness exceeding theoretical Hashin‐Shtrikman bound upper alongside dramatic strength and energy absorption enhancements versus conventional lattices. These hybrid metamaterials deliver tailored mechanical responses for biomedical implants, acoustic isolation, and adaptive infrastructure systems.

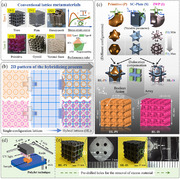

## 1 Introduction

Metamaterials, characterized by complex lattice topologies and lightweight, customizable, superior mechanical properties, are extensively used in various fields such as soft robotics^[^ ^1^ ^]^ scaffolds for tissue engineering,^[^ ^2^ ^]^ transportation,^[^ ^3^, ^4^ ^]^ and civil engineering.^[^ ^5^, ^6^ ^]^ These materials are classified into truss‐based lattices,^[^ ^7^, ^8^ ^]^ plate‐based lattices,^[^ ^9^, ^10^ ^]^ and shell‐based lattices.^[^ ^11^, ^12^ ^]^ Among these, simple cubic (SC)‐plate lattices, formed by interconnecting plates in 3D, typically exhibit higher modulus of elasticity and yield strength,^[^ ^13^, ^14^ ^]^ gaining considerable attention over the past decade.^[^ ^9^, ^15^ ^]^ However, the overhanging features of plate lattices pose challenges for additive manufacturing, often requiring support structures. Moreover, the mechanical tunability of plate lattices is inherently limited by their topological and morphological rigidity.^[^ ^16^, ^17^ ^]^ Merely providing stable load support is insufficient for metamaterials. For instance, in bone implantation applications, the stiffness must be tailored to match the surrounding tissue to avoid stress shielding. Thus, an ongoing challenge in the application of lattice metamaterials is enhancing stiffness while simultaneously ensuring tunability, crucial for widespread and flexible use.^[^ ^18^ ^]^

As the field evolves, triply periodic minimal surface (TPMS)‐based lattices can be generated using implicit methods, offering enhanced modeling flexibility. Furthermore, their intrinsic self‐supporting geometry simplifies the additive manufacturing process, reducing structural defects associated with support removal. In addition, the unique zero‐curvature surfaces of such lattices significantly reduce stress concentrations, thus preventing global failure before the structure reaches the densification stage. These advantages contribute to the wide‐ranging application prospects of TPMS across various fields. For instance, TPMS structures such as Schwarz‐P and Schwarz‐D, known for their minimal surface area under periodic boundary conditions, are considered ideal candidates for metamaterials with excellent mechanical, thermal, and electrical properties.^[^ ^19^, ^20^ ^]^ Moreover, Kelly et al.^[^ ^21^ ^]^ simulated the mechanical properties of mimicked natural bone by tailoring the porosity and elastic constants of TPMS. These studies demonstrate that TPMS, controlled by mathematical equations, can be precisely and controllably customized to meet diverse application requirements. However, existing research predominantly focuses on topological transformations within the same lattice category, neglecting the significant potential benefits of integrating multiple lattice categories. This oversight suggests a critical gap in the current understanding of lattice metamaterials. Therefore, developing advanced design strategies that incorporate multi‐category lattice integration is essential for enhancing the mechanical performance and application versatility of these materials.

Hybridization of disparate lattice topologies emerges as a transformative frontier in metamaterial engineering, where strategic integration of architectural motifs unlocks unprecedented multifunctionality beyond monolithic designs.^[^ ^22^ ^]^ For instance, Ye et al.^[^ ^23^ ^]^ hybridized truss lattices with TPMS lattices to create a novel lattice structure that integrates load‐bearing and thermal management functionalities. Similarly, Li et al.^[^ ^24^ ^]^ employed a synergistic mechanism of strong‐weak coupling to construct a hybrid lattice structure with enhanced sound absorption and energy absorption properties. Advances in additive manufacturing (AM) technology have significantly improved material precision and printing efficiency, enabling the fabrication of complex metamaterials and opening new avenues for innovation.^[^ ^25^, ^26^, ^27^, ^28^, ^29^ ^]^ Specifically, these include the hybridization of body‐centered cubic (BCC) and face‐centered cubic (FCC) lattices,^[^ ^30^ ^]^ truss and plate lattices,^[^ ^31^ ^]^ and integrating various triply periodic minimal surface (TPMS) lattices through the sigmoid function.^[^ ^32^ ^]^ Despite these advances, limited studies have focused on hybrid structures combining TPMS and plate lattices. Consequently, the exploration of stiffness and tunability potential in hybrid lattices has been further neglected.

In this work, we propose a design strategy that integrates topological dislocation compensation with Boolean fusion to develop hybrid TPMS–plate lattice metamaterials. These structures combine tunable behavior with enhanced mechanical robustness. Specifically, two configurations are introduced: HL‐PS, integrating Primitive with SC‐plate lattices, and HL‐IS, combining I‐Wrapped Package (IWP) with SC‐plate lattices. These designs leverage the curvature‐driven tunability of TPMS structures and the stress‐channeling rigidity of plate lattices through a coordinated 3D topology. To enhance the accuracy of the AM process, eight holes were introduced in the substrate plates to ensure connectivity across the assembled lattice array. Asymptotic homogenization theory was applied to efficiently compute the elastic properties of the proposed hybrid metamaterial. Subsequently, the structures were fabricated using PolyJet AM, and quasi‐static compression tests were performed on the printed samples to investigate their mechanical response. Additionally, numerical simulations were conducted to analyze the energy absorption mechanisms, which were validated by comparison with experimental results. This framework introduces a novel design paradigm in architected metamaterials, integrating mechanical robustness with adaptive functionality.

## 2 Generation of Hybrid TPMS‐Plate Lattice Structures

**Figure 1:**
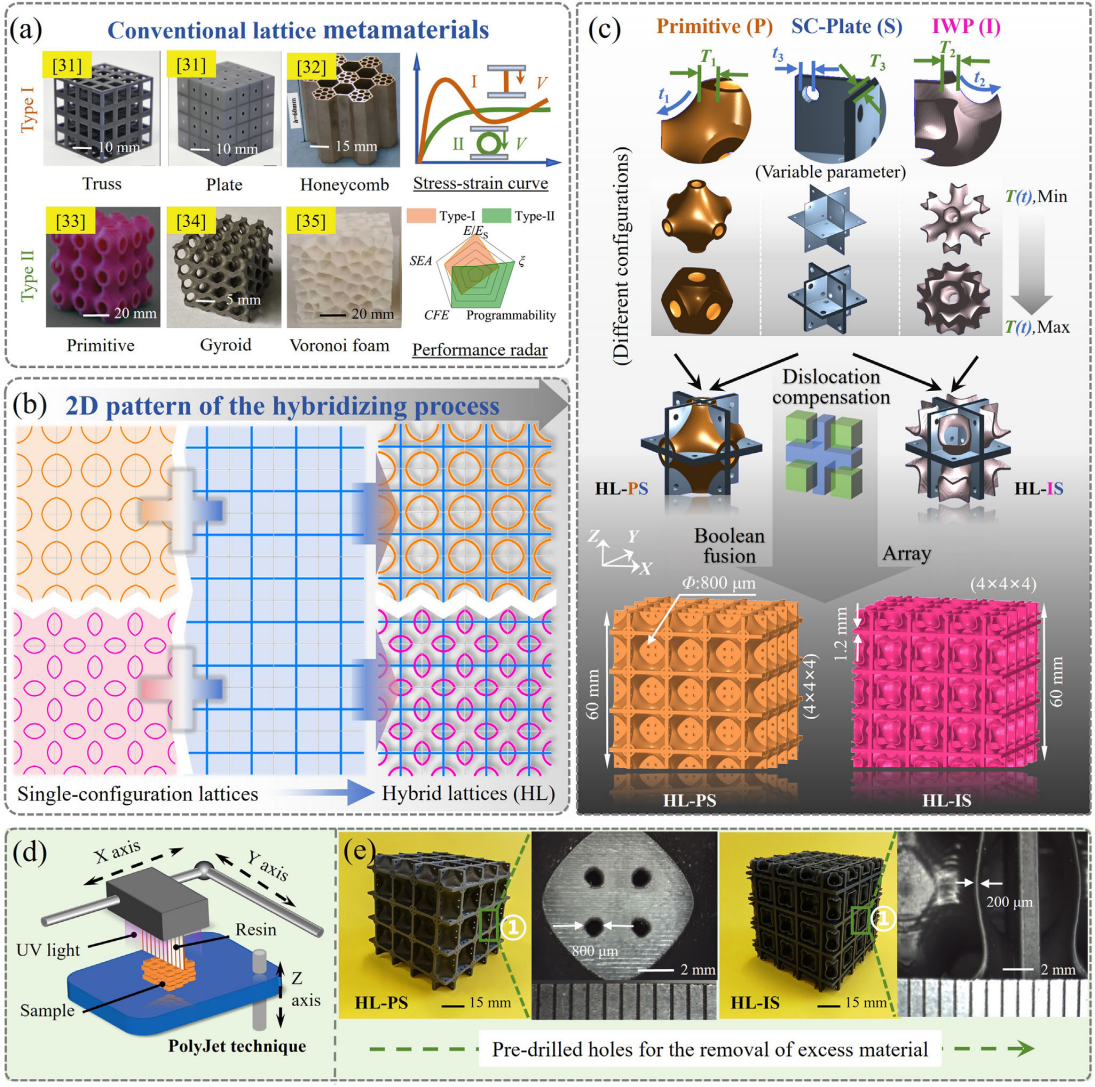
The design origin and strategy. a) Two typical conventional lattices. Type I: Truss lattice, plate lattice: Reproduced with permission.^[^ ^33^ ^]^ Copyright 2021, Elsevier. Honeycomb lattice: Reproduced with permission.^[^ ^34^ ^]^ Copyright 2023, Elsevier. Type II: Primitive lattice: Reproduced with permission.^[^ ^35^ ^]^ Copyright 2021, Wiley. Gyroid lattice: Reproduced with permission.^[^ ^36^ ^]^ Copyright 2022, Elsevier. Voronoi foam: Reproduced with permission.^[^ ^37^ ^]^ Copyright 2023, Elsevier. b) Simplified illustration of the development of single‐type lattices into hybrid lattices. c) Specific design flow and variable parameter space for hybrid lattices. d) Fabrication techniques for hybrid lattice samples. e) Topography views of the region ① of hybrid lattices under an optical microscope.

Lattice metamaterials composed of a single topological type often face challenges in independently optimizing nonlinear mechanical properties due to their interdependent constraints. For example, the design of metamaterials has been dominated by periodic strut‐based (Type I)^[^ ^33^, ^34^ ^]^ and curve‐based (Type II)^[^ ^35^, ^36^, ^37^ ^]^ architectures, as shown in **Figure** **1**a, which are constrained in balancing stiffness, strength, and stability. Calladine^[^ ^38^ ^]^ highlighted these challenges by comparing straight struts and circular beams, noting from the stress‐strain curves in Figure 1a that circular beams, while providing more stable stress fluctuations due to their initial curvature, suffer from lower stiffness and strength. Similarly, SC‐plate lattices, while stiffer than TPMS lattices of equivalent mass,^[^ ^39^ ^]^ offer limited adaptability to varying design requirements. Although TPMS structures offer considerable geometric tunability by adjusting implicit function parameters to achieve truss‐like, hollow truss‐like, or shell‐like forms, this flexibility alone is insufficient to resolve the inherent trade‐off between stiffness and tunability. Overcoming this trade‐off is therefore key to fully realizing the potential of architected metamaterials in adaptive and application‐specific contexts.

We propose a hybrid approach that combines the advantages of different lattice types to overcome the trade‐offs in single‐topology systems. Specifically, we propose two types of hybrid TPMS‐plate lattice structures, HL‐PS and HL‐IS, which integrate spatial compensation with Boolean fusion, as illustrated in Figure 1b. HL‐PS combines the Primitive lattice with the SC‐plate lattice, while HL‐IS integrates the IWP lattice with the SC‐plate lattice. Figure 1c details the construction process for these hybrid designs, and further customization options, such as unit cell variations controlled by specific parameters, are provided in Section 1 and Table S1 (Supporting Information).

The hybrid design leverages the SC‐plate lattice as a structural skeleton to provide high stiffness and strength while incorporating the TPMS lattice to absorb stress and enhance design flexibility. This combination achieves a balance between mechanical performance and tunability, addressing the challenges of independently optimizing nonlinear mechanical properties. Additionally, the Boolean fusion technique ensures seamless integration at the interfaces, reducing stress concentrations and improving load distribution across the structure (Section 2, Supporting Information). These hybrid structures not only overcome the inherent trade‐offs in single‐topology designs but also open up new possibilities for advancing applications.

In our work, Equations (1) and (2) detail the two TPMS implicit functions used to define surfaces within the TPMS framework. The solid model of the isosurface is defined by Equation (3), where the shell thickness of the TPMS is adjusted via *T_1_* or *T_2_* to determine the relative density of individual cells.

Primitive

(1) $$\phi_{P}\left(x,y,z\right)=\cos\left(\omega x\right)+\cos\left(\omega y\right)+\cos\left(\omega z\right)$$

IWP

(2) $$\begin{array}{c} \phi_{IWP}\left(x,y,z\right)=\left[\cos(\omega x)\cos(\omega y)+\cos(\omega y)\cos(\omega z)+\cos(\omega z)\cos(\omega x)\right]\\ -0.5\left[\cos(2\omega x)+\cos(2\omega y)+\cos(2\omega z)\right]+t_{2} \end{array}$$

TPMS Lattice

(3) $$\phi_{solid}\left(x,y,z\right)=\left[\phi_{i=P,IWP}\left(x,y,z\right)+{T_{i}}_{=1,2}\right]\left[\phi_{i=P,IWP}\left(x,y,z\right)-T_{i=1,2}\right]$$

Here, *x*, *y*, *z* represent spatial coordinates, with *ω* = 2π/*l*, where *l* is the length of the cell. The values *t*_1_ and *t*_2_ from Equations (1) and (2) set the level sets, controlling the shapes of the Primitive and IWP lattices, respectively. For TPMS lattices that cannot be continuously produced or do not meet the minimum thickness requirements for additive manufacturing, we restrict parameter ranges to ensure only structures that conform to the lattice morphology and fabrication process requirements are generated.^[^ ^40^ ^]^ Additionally, SC‐plate lattices are uniform plates controlled by thickness *T*_3_ and radius *t*_3_. The design includes circular holes in each plate primarily to enable the removal of internal support material during PolyJet 3D printing. Fully enclosed cavities would trap support material, increasing part weight and compromising structural integrity. These vents allow complete drainage and dissolution of supports. Importantly, the intrinsic self‐supporting nature of TPMS surfaces mitigates the need for additional supports during fabrication, effectively addressing the challenge posed by overhanging components in SC‐plate lattices.

The structures designed in this study were manufactured using the PolyJet technique on a 3D printing machine (SimpNeed P300), utilizing a resin‐based Polycarbonate composite polymer (PC+), as depicted in Figure 1d. The detailed operations are described in the Experimental Section. Notably, two types of hybrid lattice structures, HL‐PS and HL‐IS, were prepared, with a minimum channel size of 800 µm, ensuring the egress of excess material through the apertures. Due to boundary effects, the number of unit cells in a sample may influence its mechanical response. Research indicates that a 4 × 4 × 4 lattice arrangement sufficiently characterizes mechanical properties, balancing cost and the resemblance to an infinite array.^[^ ^41^, ^42^, ^43^, ^44^ ^]^ The lattice samples studied had dimensions of 60 mm × 60 mm × 60 mm, are shown in Figure 1e. To ascertain the material properties of the PC+ substrate employed in this research, tensile tests on dog‐bone specimens were performed according to ASTM D638 standards^[^ ^45^ ^]^ (Section 4, Figure S3, Supporting Information).

## 3 Results and Discussion

### 3.1 Tunable Mechanical Properties of Hybrid Structures

This study employs voxel‐based homogenization theory to efficiently compute the effective elastic properties of lattice structures, thereby optimizing computational resources. The 64 × 64 × 64 resolution aligns with established practices for lattice analysis.^[^ ^6^ ^]^ For 3D orthotropic materials, Young's modulus (*E*), and the Zener anisotropy ratio (*Z*) for evaluating the anisotropy can be computed through homogenization methods (Section 3, Supporting Information). Subsequently, the study analyzed the effective elastic properties of the original lattices, including two types of TPMS (Primitive and IWP), and SC‐plate lattices featuring pre‐set holes, as along with the novel TPMS‐plate lattices.

#### 3.1.1 Stiffness Analysis of the Lattice Structures

To systematically evaluate the mechanical performance of the lattices, the normalized Young's modulus of each architecture was compared against relevant theoretical bounds. The Hashin‐Shtrikman (HS) upper bound^[^ ^46^ ^]^ (*E*_HSU_) represents the theoretical maximum stiffness that cellular solids can achieve, as defined by Equation (4).^[^ ^46^ ^]^

(4) $$E_{HSU}=\frac{2E_{S}\bar{\rho }\left(7-5v_{s}\right)}{15\left(\bar{\rho }-1\right)v_{s}^{2}+2\left(\bar{\rho }-6\right)v_{s}-13\bar{\rho }+27}$$

Here, *E*_s_ and versus represent the modulus of elasticity and Poisson's ratio of the solid material, respectively, and $\bar{\rho }$ denotes the relative density of the metamaterials.

Additionally, Zhong et al.^[^ ^47^ ^]^ proposed an extended Gibson‐Ashby model (denoted as *E*_EGA_) based on the classical Gibson‐Ashby framework, originally developed for deformation dominated by a single mechanism. The extended model incorporates the combined effects of stretching, bending, and shear deformation mechanisms, providing a more comprehensive prediction of the elastic modulus under multi‐mode deformation scenarios, with the formulation expressed as follows:

(5) $$E_{EGA}=\frac{{\bar{\rho }}^{2}}{0.7+3.8\bar{\rho }}E_{s}$$

**Figure 2:**
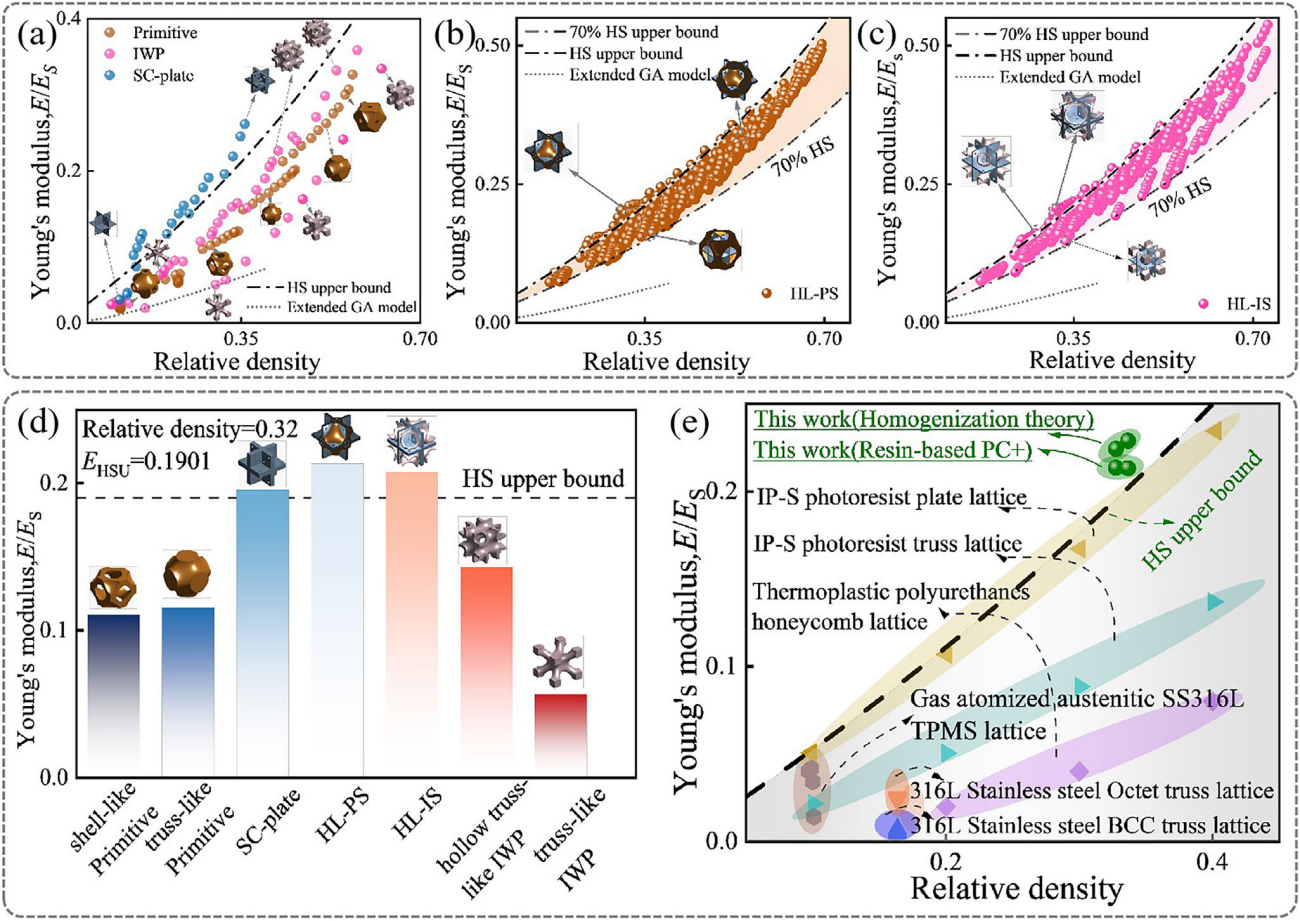
The normalized Young's modulus of the lattice structures. a) Original lattices, b) HL‐PS, c) HL‐IS, and d) Comparison with HS upper bound. (The black dashed line shows the HS upper bound for isotropic stiffness under 0.32 relative density), e) Ashby maps depicting the normalized Young's modulus of the TPMS‐plate lattices and previously proposed lattices.

Previous studies have confirmed that SC‐plate lattices possess outstanding stiffness, with *E*/*E*_S_ values that exceed the edges of the HS upper bound,^[^ ^14^, ^16^ ^]^ as illustrated by the blue scatter data in **Figure** **2**a. Furthermore, the pre‐drilled holes in the plate appear to have minimal impact on overall performance.^[^ ^48^ ^]^ By adjusting shape and thickness parameters, a single TPMS model can generate a continuum of lattice structures with varying topologies, including truss‐like, hollow truss‐like, and shell‐like configurations. As the relative density exceeds 0.3, the *E*/*E*_S_ for both truss and hollow truss‐like Primitive structures become uniformly distributed, as shown in Figure 2a, with minimal differences between them. This indicates that the configuration of Primitive lattices has little effect on mechanical properties across a broad range of densities. On the other hand, the IWP can form both truss and hollow truss‐shaped configurations. Among the tested samples, truss‐like IWP structures exhibit the lowest *E*/*E*_s_), indicating lower stiffness compared to Primitive with similar solid configurations. This phenomenon is attributed to the bending‐dominated characteristics of IWP structures. Generally, structures dominated by stretching tend to have higher stiffness as they directly resist applied loads. However, an interesting exception occurs with bending‐dominated, hollow truss‐like structures formed by the IWP, which surpasses the stiffness of solid truss, stretching‐dominated Primitive structures. This finding indicates that the hollow truss‐like IWP lattices not only reduce material usage and enhance energy dissipation but offer superior stiffness.

This diversity, combined with a hybridization strategy, enables the design of TPMS‐plate metamaterials with adjustable shapes and sizes. In particular, the HL‐PS structure combines shell‐like and plate lattices, while HL‐IS combines truss‐like and plate lattices, representing the configurations with the lowest stiffness in the dataset. Among the hybridized lattices, HL‐PS, which combines a low‐density hollow truss‐like with a plate lattice, achieves an *E*/*E*_S_ up to 157.55% of the HS upper bound. Similarly, within the HL‐IS structures, the maximum *E*/*E*_S_ surpasses the HS upper bound, reaching up to 120.84%. Figure 2b,c demonstrates that the *E*/*E*_S_ of TPMS‐plate lattice structures, determined through numerical homogenization across a relative density range of 0.05 to 0.75, exhibits continuous tunability. The hybrid lattices expand the tunable range of effective elastic modulus by 213.98% compared to conventional ultrastiff plate lattices.

Figure 2d illustrates a comparison of *E*/*E*_S_ values for various lattice types at a relative density of 0.32, where the HS upper bound for Young's modulus (*E*_HSU_) is 0.1901. The truss‐like Primitive demonstrates a significant stiffness advantage over the truss‐like IWP, exceeding it by 103.98%, primarily due to the combined effects of stretching and bending mechanisms. Additionally, the hollow truss‐like IWP demonstrates a stiffness 152.77% higher than that of the truss‐like IWP. This finding emphasizes the considerable potential of incorporating hollow truss structures into hybrid lattice frameworks, thereby substantially increasing structural stiffness relative to conventional truss lattices. Notably, Zhao et al.^[^ ^49^ ^]^ proposed near‐isotropic metamaterials achieving over 98% of the HS upper bound, while Meyer et al.^[^ ^9^ ^]^ developed stochastic plate lattice topologies demonstrating stiffness superior to truss and shell architectures. Despite these advances, manufacturing constraints persist in monolithic plate lattice realization. Our work demonstrates unprecedented stiffness advantages as well as manufacturability advantages. Consequently, the stiffness of HL‐PS and HL‐IS surpasses this upper bound by 12.29% and 9.28%, respectively. Figure 2e presents a Gibson‐Ashby chart that compares the *E*/*E*_S_ of both HL‐PS and HL‐IS with recent research on various lattice structures, including typical truss, plate, honeycomb, and hybrid lattice structures.^[^ ^9^, ^48^, ^50^, ^51^ ^]^ While the extended Gibson‐Ashby model offers a suitable framework for capturing the mechanical behavior of low‐density cellular materials, our comparative analysis focuses specifically on the widely adopted HS upper bound, consistent with most previous studies. The grey shaded area in the lower right corner represents the stiffness range of cellular solid materials according to HS theory. The chart clearly illustrates the stiffness advantage of the TPMS‐plate lattice structures proposed by our work.

#### 3.1.2 The Degree of Anisotropy of the Lattice Structures

Research suggests that the stiffness of plate lattice structures and TPMS lattice structures is highly dependent on the direction of the applied load.^[^ ^52^ ^]^ In specific load directions, the stiffness varies due to the stress distribution, with greater stiffness typically observed in directions aligned with the support configuration. Under variable or unknown load conditions, isotropic material properties offer distinct advantages.^[^ ^53^ ^]^ For instance, at the macroscopic level, first‐generation porous isotropic materials, such as random solid foams, are widely used for elastic buffering and impact energy absorption. The anisotropy of lattice structures is closely tied to the arrangement of their features. In this section, we apply homogenization techniques and use MATLAB to visualize the elastic modulus of the lattices in 3D space. Moreover, since all the lattices in the present study exhibit cubic symmetry, we employ Zener anisotropy ratio (*Z*), defined as *Z = 2G(1+ν)/E*, to quantify and compare the degree of isotropic across different lattice types at various relative densities. Here, *G*, *ν*, and *E* represent the shear modulus, Poisson's ratio, and Young's modulus of the lattice structures, respectively.

**Figure 3:**
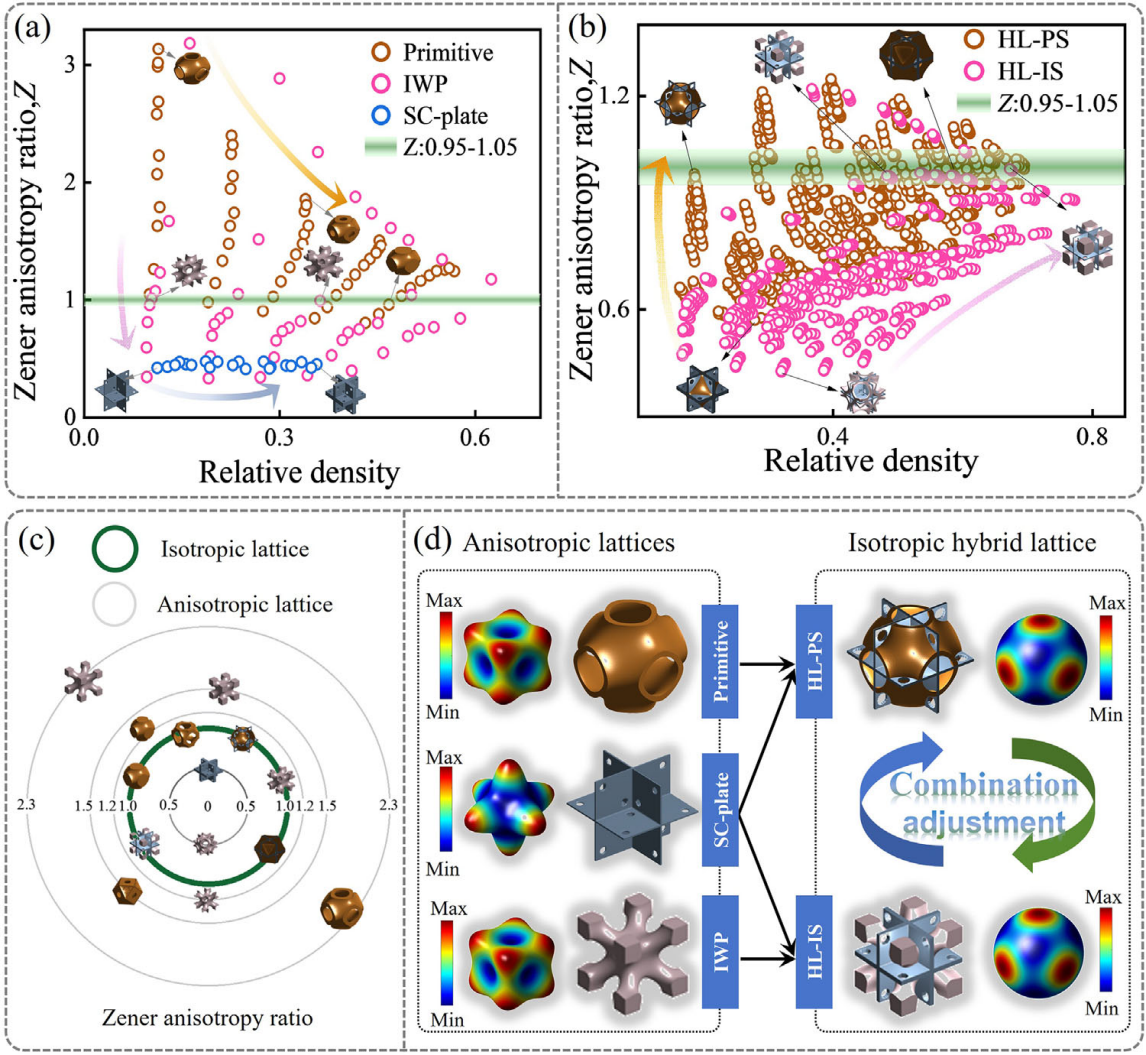
Comparison of Zener ratio between lattice structures. a) Original lattices. b) TPMS‐plate lattices. c) Zener ratio map of original lattices and TPMS‐plate lattices. d) The effective Young's modulus surfaces of the lattice structures.

**Figure** **3**a conducts a comparative analysis of isotropic elasticity in SC‐plate and TPMS lattices. It has been observed that among the three original lattice types, the near‐spherical solid geometry of Primitive promotes a consistent distribution of material properties throughout all spatial dimensions, thereby enhancing their isotropic behavior. Furthermore, IWP lattices leverage their traditional truss‐like and hollow truss‐like configurations to markedly diminish anisotropy as transitioning from solid to hollow struts. Meanwhile, the Z of SC‐plate lattices typically remains ≈0.5, failing to achieve isotropic elasticity. Figure 3b conducts a comparative analysis of the elastic anisotropy in TPMS‐plate lattice structures. Most Z data points for HL‐IS structures fall below 1, while those for HL‐PS structures are closer to 1, indicating that the latter are closer to achieving isotropy.

Figure 3c visually presents lattice examples under different *Z* values using concentric circles, where the radius signifies the *Z* value, and a bold green line indicates isotropic lattices with a *Z* of 1. Additionally, Figure 3d displays the effective Young's modulus surfaces of the lattice structures. In these surfaces, the color spectrum ranges from blue to red, denoting the minimum to maximum Young's moduli, respectively. For example, the elastic surface is more dispersed in the truss‐like IWP lattices, a stark contrast to the SC‐plate lattices, where lattice materials are relatively dependent of axial supports. Isotropy is significantly enhanced as the *Z* approaches 1, as seen in the varied elastic surfaces. Specifically, the uniform characteristics of certain TPMS lattices effectively neutralize the pronounced directional properties of plate lattices, resulting in a more consistent response across all directions in hybrid lattice structures. For instance, combining plate elements with a *Z* of 0.47 with Primitive elements having a *Z* of 3.14 achieves an overall *Z* of 1. Similarly, the combination of truss‐like IWP elements with plate elements also satisfies isotropic characteristics, underscoring the crucial role of structural arrangement and component interaction in achieving optimal mechanical responses. This design achieves a more uniform distribution of stress among the supporting elements during deformation, thereby enhancing the overall uniformity of load resistance.

Historically, research has often achieved isotropic lattice structures by combining varying proportions of Simple Cubic (SC), Body‐Centered Cubic (BCC), and Face‐Centered Cubic (FCC) structures within a single unit cell.^[^ ^16^, ^48^, ^54^, ^55^ ^]^ However, to the best of the authors' knowledge, this is the first report of isotropic geometries induced by compensatory hybridization of SC‐plate and TPMS unit cells. While individual TPMS lattices and isotropic support structures also exhibit near‐isotropic behavior, the elastic moduli of these lattices are significantly lower than those of the proposed TPMS‐plate lattices. Our work can contribute to the advancement of metamaterials with tunable mechanical properties, suitable for a broad range of applications.

### 3.2 Quasi‐Static Compression Behavior

#### 3.2.1 Comparison of Hybrid Lattices

**Figure 4:**
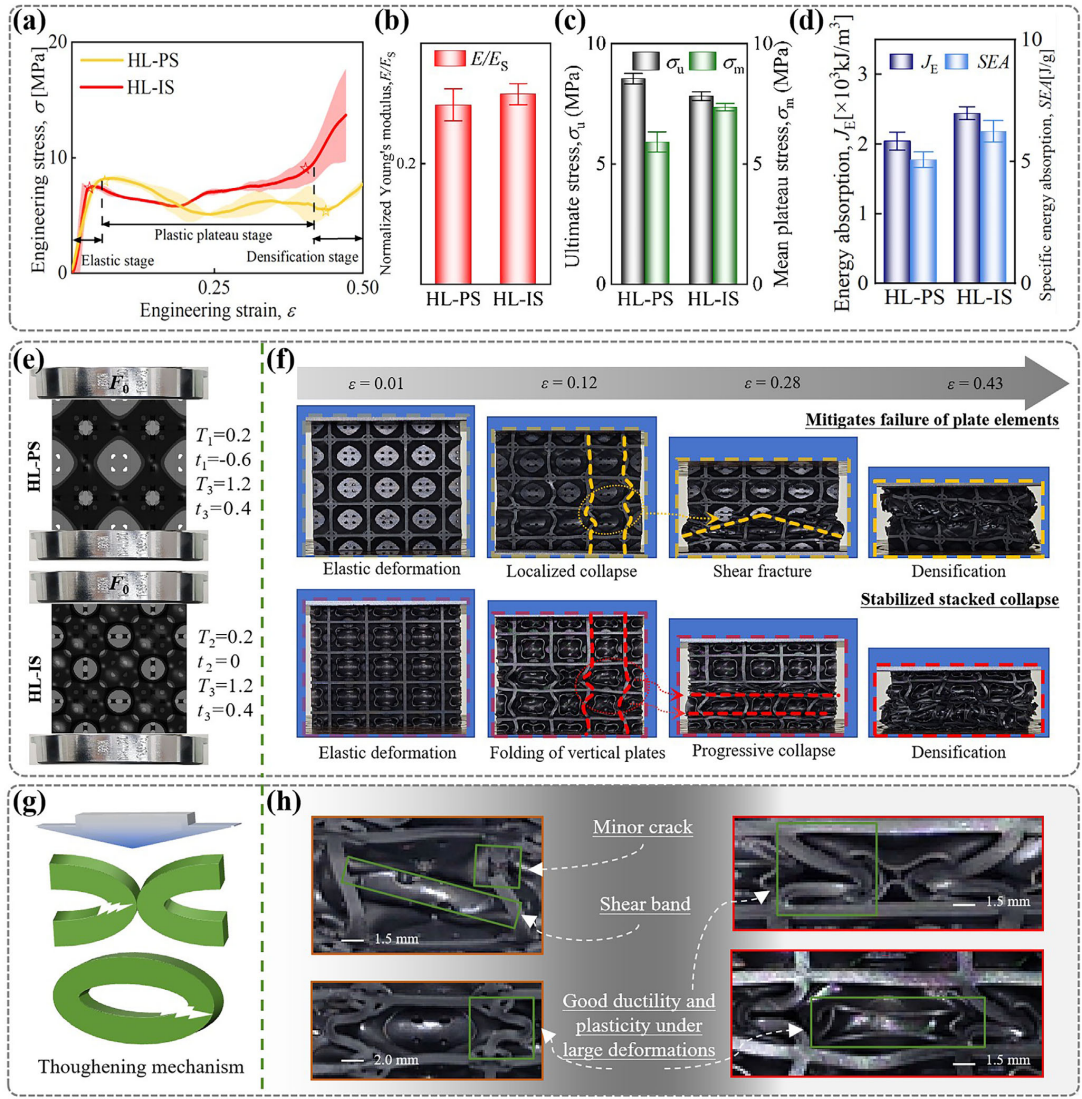
Compression results for hybrid lattices. a) Engineering stress‐strain curves. b) Normalized Young's modulus. c) Initial ultimate stress σ_u_ and mean plateau stress σ_m_. d) Energy absorption J_E_ and specific energy absorption SEA. e) Initial state of samples for quasi‐static compression tests. f) Compressive deformation modes of hybrid lattices. g) Schematic diagram of toughening mechanism. h) Details of localized deformation.

To further validate the design concepts in this work, we investigated the mechanical response under quasi‐static compression for the HL‐PS and HL‐IS structures. We conducted four sets of compression tests using a universal testing machine to obtain stress‐strain curves, as shown in **Figure** **4**a. Results indicate high consistency between two specimens within the same group, confirming the reliability of AM technology in producing hybrid lattice structures with controllable properties.

The stress‐strain curves reveal that the compression deformation of both structures under constant‐rate quasi‐static compression can be categorized into three typical stages: I) elastic, II) plastic plateau, and III) densification stages. Nominal strain, *ε* (the ratio of compression displacement to the initial length of the structure), quantitatively describes the relative deformation of the specimens. As depicted in Figure 4f, the deformation modes of both structures are delineated using yellow or red dashed frames. The vertical plate elements of HL‐PS exhibit a pronounced tendency to fold at the outer layer, with less folding in the inner layer (yellow dashed line, *ε* = 0.12). This discrepancy is accompanied by the formation of an inverted “V”‐shaped shear band as the collapse progresses, as indicated by the yellow dashed line (*ε* = 0.28). This localized shear deformation does not result in global failure, instead, it leads to a pyramid‐like configuration in the mid‐axis region, where mutual extrusion further enhances structural support. As depicted in the stress‐strain curves in Figure 4a, after reaching an initial peak stress, the HL‐PS experienced a slight reduction in stress, followed by a phase of secondary hardening. In contrast, HL‐IS does not display distinct shear fracture bands, instead, it shows a deformation pattern where the plate elements fold uniformly (red dashed line, *ε* = 0.12), resulting in “I”‐shaped compression bands at various axial locations (red dashed line, *ε* = 0.28). Ultimately, as the deformation bands expand and accumulate, lattice structures progressively collapse and exhibit lateral expansion, resulting to a rapid increase in load upon material contact.

Overall, both proposed hybrid lattices demonstrate asymptotic collapse modes and sustain stable plastic deformation without abrupt fractures leading to global collapse. Figure 4h illustrates the presence of minor cracks in the HL‐PS structure during compression, indicating both structures maintain good ductility and plasticity under large deformations. Details of local deformation reveal the toughening mechanism of hybrid lattices (Figure 4g). White zigzag arrows indicate stress‐concentrated zones susceptible to crack initiation. Furthermore, no interfacial tearing occurs between the plate elements and TPMS elements during deformation, confirming the effectiveness of the Boolean fusion strategy implemented at the design stage (Supporting Information), which successfully prevents issues of poor interfacial connectivity between elements.

Figure 4b illustrates the values of the normalized Young's modulus (*E*/*E*_S_) for the structures. HL‐PS achieves a *E*/*E*_S_ of 0.2243 MPa at a relative density of 0.33, representing 137.34% of the HS upper bound. In contrast, HL‐IS demonstrates a *E*/*E*_S_ of 0.2289 MPa at a relative density of 0.34, which surpasses the HS upper bound by reaching 110.84% of this limit. Experimental results show less than 7% errors from the E/Es values presented in Section 3.1.1, confirming the predictive accuracy of the homogenization theory framework. The results demonstrate that the proposed hybrid lattices can achieve ultrahigh stiffness, benefiting from the role of the SC‐plate lattice as a skeleton support for the sub‐lattice. Moreover, its orthorhombic isotropic stiffness is unprecedented among the lightweight samples that can currently be fabricated. Further quantitative comparison of the plastic behavior of hybrid lattices, key mechanical indicators such as ultimate stress (*σ*_u_), mean plateau stress (*σ*_m_), energy absorption per unit volume (*J*_E_), and specific energy absorption (*SEA*) are calculated. Definitions for these terms are outlined in the Experimental Section. The material distribution of the TPMS element in HL‐PS is concentrated near the mid‐axis, forming a central‐supported skeletal structure. This configuration results in an initial ultimate stress *σ*_u_ that is 9.29% higher than that of HL‐IS. In the subsequent plastic phase, HL‐IS exhibits enhanced toughness, characterized by excellent ductility and plasticity in a progressive collapse mode. It also demonstrates a heightened capacity for plastic energy dissipation prior to densification. Specifically, the *σ*_m_, *J*_E_, and *SEA* in HL‐IS are 19.70%, 19.47%, and 15.98% higher, respectively, compared to HL‐PS, as shown in Figure 4c,d. Consequently, TPMS elements with curvature demonstrate varying performance contributions based on their topological configurations.

#### 3.2.2 Mechanical Comparison and Enhanced Mechanisms

We employ numerical simulations to compare the crushing behavior of the two types of TPMS‐plate lattices with their original lattice counterparts. This comparison aims to further explore the mechanical advantages and potential energy absorption mechanisms of the hybrid designs. The finite element models are described in the Experimental Section. The material property of the structure was modeled as ideal elastoplastic solids, with the simulated material characteristics defined by the true stress‐strain curve (Section 4, Figure S3, Supporting Information). The comparison of the stress‐strain curves and deformation processes between the experimental and simulated results reveals satisfactory agreement, validating the reliability and accuracy of the finite element models (Section 6, Supporting Information).

**Figure 5:**
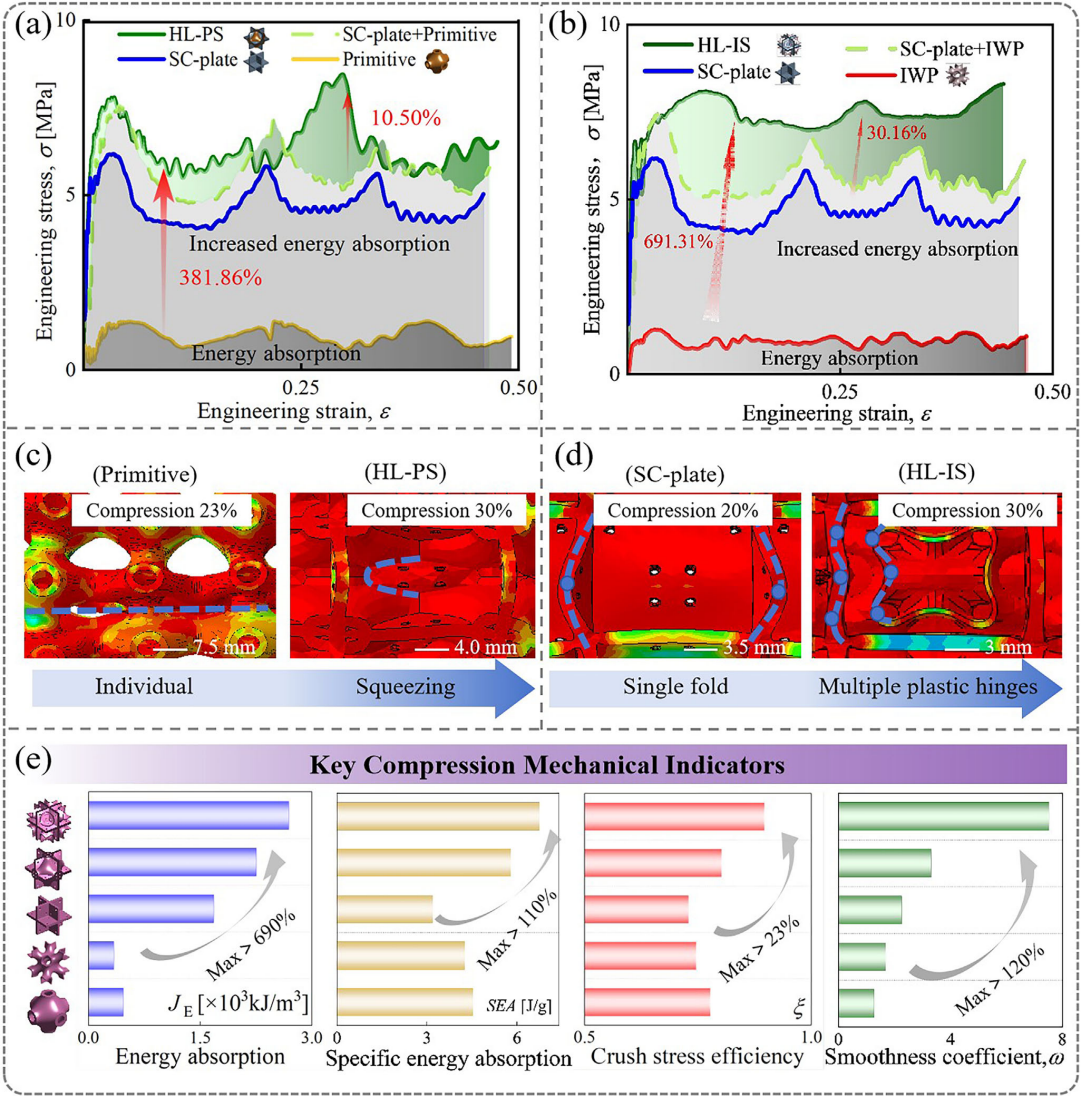
Comparison of original lattices and hybrid lattices. a) Force‐displacement curves of the corresponding individual components and HL‐PS. b) Force‐displacement curves of the corresponding individual components and HL‐IS. c) Localized deformation of single lattice and HL‐PS. d) Localized deformation of single lattice and HL‐IS. e) Key mechanical indicators.

This section begins by analyzing the Primitive, SC‐plate, and HL‐PS structures individually. Deformation patterns for all lattice structures are provided for Section 6, Figure S6 (Supporting Information). As illustrated in **Figure** **5**b, the Primitive lattice features intermediate layers that initially form a single compression band without shear or relative sliding, showcasing a characteristic pattern of gradual stress evolution pattern. The manifestation is shown in the stress‐strain curve remains stable with minimal fluctuations during the plastic plateau phase, as illustrated in Figure 5a. Conversely, the vertical elements of the SC‐plate lattice structure tend to fold under compression, forming “X”‐shaped shear band. This folding results in significant fluctuations in the stress‐strain curve during the plastic plateau phase, with peaks and valleys corresponding to the folding deformation, as shown in Figure 5a. To clearly illustrate the advantages of HL‐PS, independent stress‐strain curves for all three types of lattices are presented. Additionally, the total stress‐strain curve for HL‐PS is compared to the combined curves of the Primitive and SC‐plate lattices.

**Figure 6:**
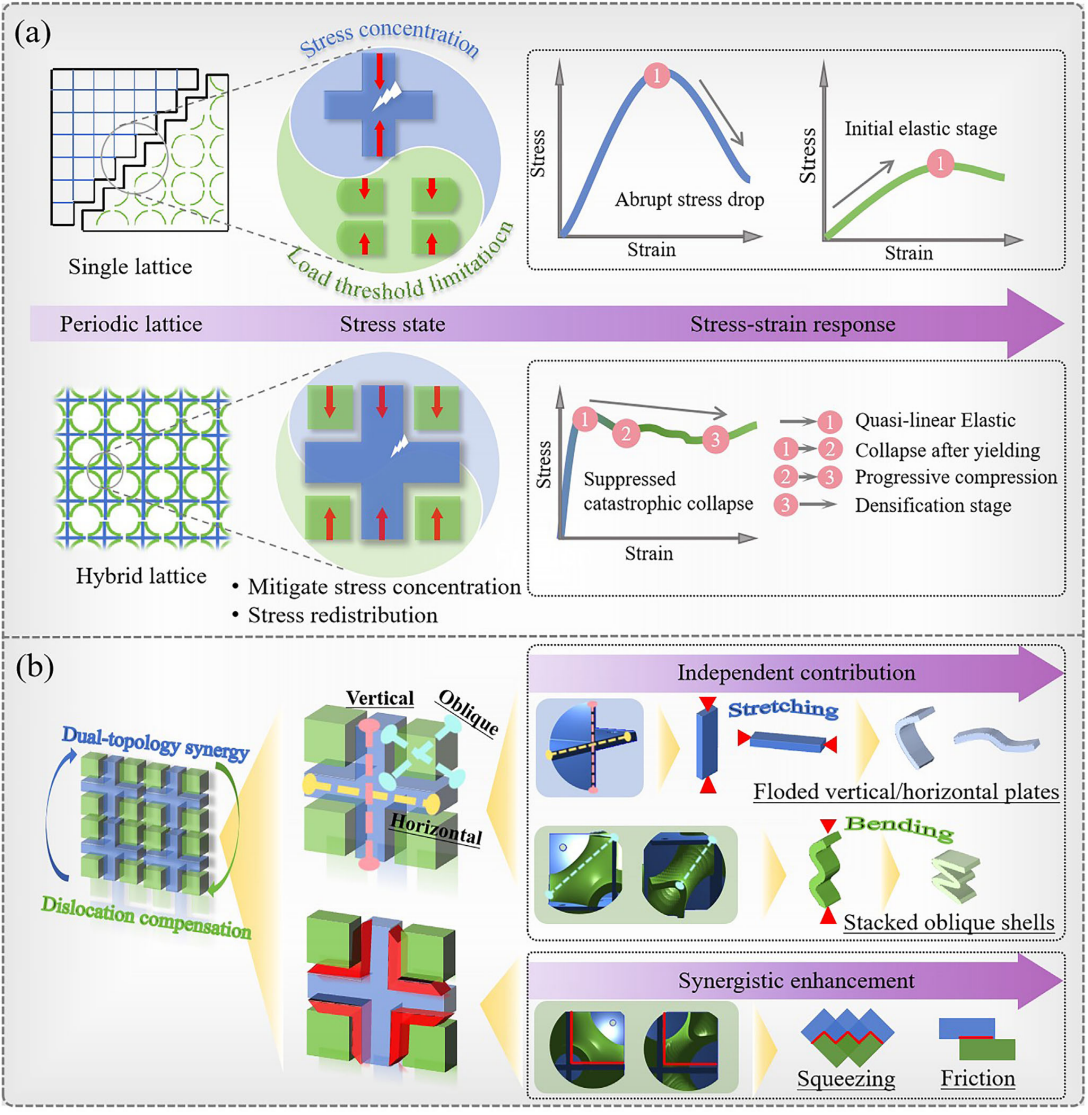
Mechanical mechanism analysis. a) Schematic diagram of compression curves corresponding to different failure modes. b) Synergistic mechanism of hybrid lattices.

In the initial elastic region, the stiffness of the hybrid lattice (HL‐PS) shows only minimal differences compared to the SC‐plate lattice, which still maintains a significantly ultrastiffness, as discussed in the previous Section 3.1.1. **Figure** **6**a,b schematically illustrate the mechanism behind the crush response and deformation patterns of the conventional single lattices and the proposed hybrid lattices propose. The external load applied to the plate lattice directly compresses both vertical and horizontal elements, and the lack of cushioning leads to a lower damage threshold at the plate junctions—visually represented by prominent white zigzag arrows indicating high‐stress fracture zones. In contrast, the hybrid lattice reduces localized stress concentration at the intersections of the separating plates through the incorporation of TPMS. This mitigation effect is abstractly denoted by significantly smaller white zigzag symbols in corresponding regions (Figure 6a), visually reinforcing how the TPMS phase disperses stress and suppresses crack initiation. Moreover, the lattice structure with dual topology features has horizontal, vertical plate, and oblique shell elements, allowing the structure to exhibit both bending and stretching mechanical behaviors, as shown in Figure 6b. The design optimizes the stress transfer paths between elements, enabling more efficient energy dissipation. In addition, within the constrained space, TPMS effectively enlarges the contact area between cells through a dislocation compensation layout in the blank quadrant of the SC‐plate lattice. This enhancement improves the capacity to dissipate energy through mutual squeezing and friction. The green area in Figure 5a highlights the performance improvements due to hybridization, showing that the energy absorption capacity of the HL‐PS is 10.50% higher than the sum of the individual lattices.

Subsequently, the nonlinear mechanical response of HL‐IS was analyzed, and the differences in deformation responses between the original IWP and SC‐plate lattices were compared. The IWP lattice displays stability during the plateau phase, with no significant peaks or troughs. In contrast, the plates in each SC‐plate cell typically fold once, creating a single plastic hinge. The HL‐IS benefits from increased stiffness due to its plate elements, while the IWP acts as a trigger for stable deformation of these plate units. As shown in the schematic Figure 6b, the oblique surface feature of TPMS suppresses buckling collapse in the support plate. The response process of structural smoothing is promoted. This is reflected in the stress‐strain curve by minimal oscillations and high strength during the plastic plateau phase, as depicted in Figure 5d. As buckling and bending deformations take place in the overall structure, the number of plastic hinges in the plate elements increases significantly (Figure 5d), enhancing both the stability under compression and the structure's ability to dissipate energy.

Figure 5b shows the stress‐strain curves for both individual and hybrid lattices. By summing the stress‐strain curves of IWP and SC‐plate lattices and comparing them with that of the hybrid structure, the mechanical superiority of the hybrid design becomes evident. The *σ*_u_ of the hybrid structure is 9.11% higher than the combined load of the individual components, mainly due to the mutual compression between IWP and the plate elements, as well as the increased number of plastic hinges. These factors significantly improve the energy absorption capacity of the hybrid structure, which is 30.16% higher than the sum of the individual IWP and plate structures.

To evaluate the energy absorption characteristics of these structures, key mechanical indicators such as *σ*_u_, *σ*_m_, *J*_E_, and *SEA*, crush stress efficiency (*ξ*), and smoothness coefficient (*ω*) are calculated (Experimental Section, Equations ((6), (7), (8), (9))). Figure 5e plots a comparison chart of these indicators for different lattices. For hybrid lattices, the energy absorption per unit volume, specific energy absorption, compression stress efficiency, and smoothness coefficient exceed those of single lattices, with maximum increases noted at 690%, 110%, 23%, and 120%, respectively. In summary, the proposed hybrid lattices, which incorporate plates and shells oriented in various directions along with synergistic interactions between cells, exhibit enhanced stability in compression deformation and an increased capacity for plastic energy dissipation. These robust features effectively mitigate the incidence of abrupt fractures in brittle materials and reduce Euler buckling.

## 4 Application Prospects

**Figure 7:**
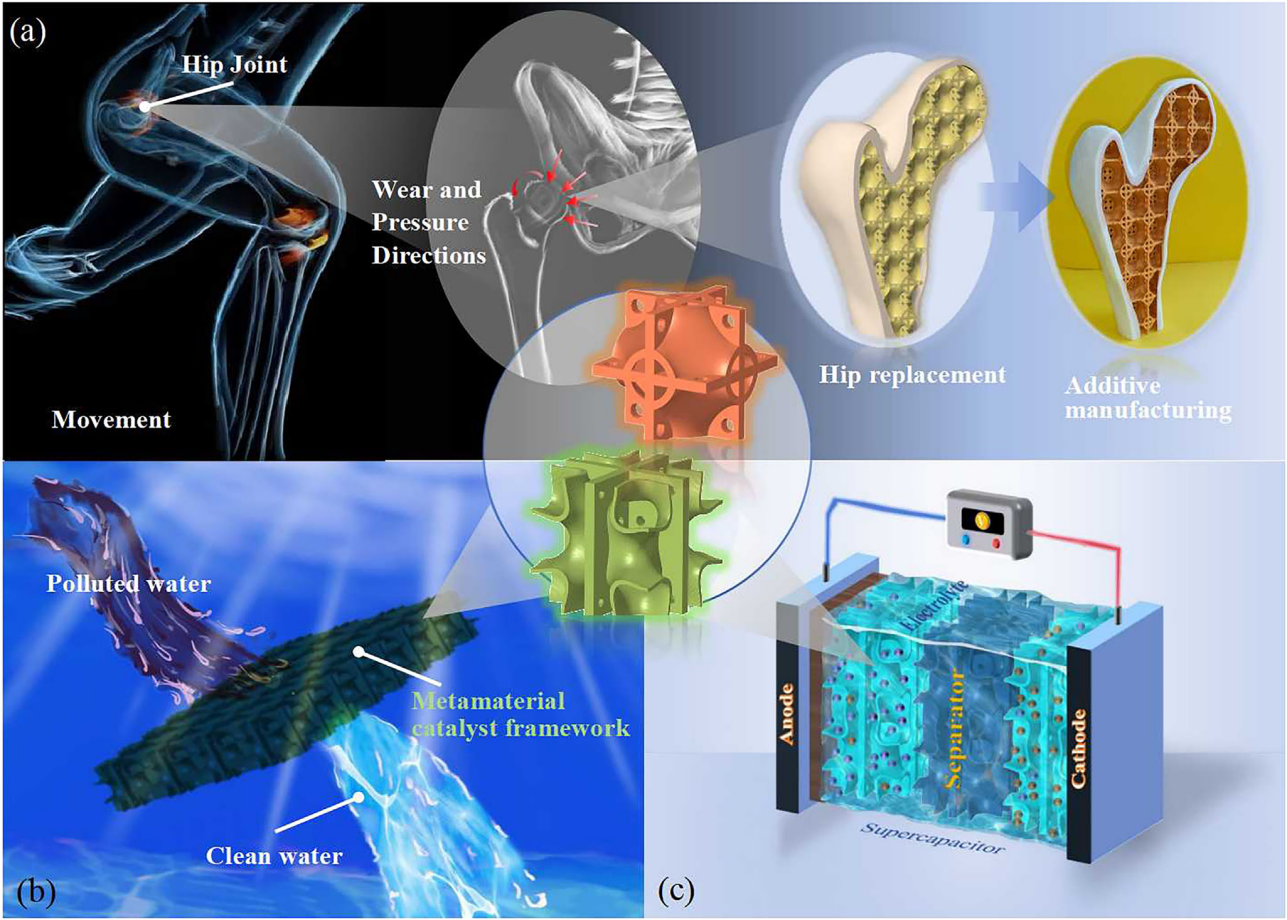
Future relevance of the proposed hybrid metamaterial for developing various applications. a) Hip replacement surgery, b) Polluted water filtration systems, c) Supercapacitor.

The multi‐topology cooperative lattice design represents a significant paradigm shift in the development of metamaterials with tunable mechanical intelligence, enabling on‐demand parameter modulation through architectural programming (**Figure** **7**). This methodology exhibits inherent scalability beyond the demonstrated SC‐plate/TPMS hybridization, as the fusion strategy can be extended to arbitrary lattice combinations (e.g., diamond cubic plates with Gyroid shells, or FCC plates with I‐WP surfaces) to create performance‐complementary architectures. Optimal bone ingrowth and cellular proliferation occur at 60% porosity, while certain materials require sub‐10% porosity to achieve the target Young's modulus.^[^ ^36^, ^56^ ^]^ Our architected scaffolds enable porosity tuning across 25%–95%, synergistically addressing dual demands: high‐porosity regions facilitate osseointegration, while low‐porosity domains achieve stiffness meeting cortical bone via material substitution.^[^ ^57^, ^58^ ^]^ This stiffness‐porosity decoupling mechanism permits effective mitigation of stress shielding through site‐specific modulus matching without compromising bioactivity.

While demonstrating isotropic material superiority, we further engineered SC‐plates into bioinspired anisotropic architectures through unidirectional rib interlacing – a topological innovation transcending conventional architectural constraints. This approach exemplifies the extensible nature of the hybridization paradigm: identical principles enable directional tuning of other lattice types (e.g., Kelvin cells or rhombic dodecahedrons) through strategic material redistribution. Drawing from nature's cellular blueprints,^[^ ^59^, ^60^ ^]^ this degradation strategy mimics bone's axial compression resistance and plant stems' lateral wind resistance, achieving a Young's modulus 129.97% above the HS upper bound specifically along the principal stress axis through material redistribution while systematically attenuating stiffness in orthogonal axes to meet directional functional priorities. These anisotropic configurations establish multifunctional synergies across engineering domains. Biomedical implants could harness directional stiffness gradients to better mimic natural tissue interfaces. Catalytic reactors might utilize programmed permeability pathways to optimize reactant flow distribution. Thermal management systems could benefit from tailorable expansion coefficients that mitigate thermal stresses in specific orientations. Such cross‐domain adaptability highlights the potential of architectured anisotropy. The bioinspired metamaterial combines 26.22% mass reduction with enhanced fluid permeability along its architectured axis, where programmed mechanical anisotropy couples with transport enhancement through morphology‐driven design, establishing multifunctionality across catalytic, electrochemical, and thermal systems.^[^ ^61^, ^62^, ^63^ ^]^

## 5 Conclusion

In this paper, two novel hybrid lattices are proposed, which integrate the Primitive, IWP lattice with SC‐plate lattices, called HL‐PS and HL‐IS, respectively. The hybrid lattices successfully address the imbalance between stiffness and tunability, with the resulting structures exhibiting high strength, toughness, and stable stress fluctuations. The study was carried out through homogenization theory, quasi‐static tests, and numerical simulations, the specific conclusions are as follows:

1. The hybrid lattices demonstrate continuously adjustable properties across a relative density range of 0.05–0.75, with a 213.98% increase in the tunable span of effective elastic modulus compared to conventional ultrastiff plate lattices.
2. Boolean fusion of SC‐plate and TPMS lattices leverages their complementary axial stiffness to achieve an isotropic Zener ratio of ≈1.
3. Experimental results show that HL‐PS and HL‐IS structures exceed the HS upper bound of normalized Young's modulus by 37.34% and 10.84%, respectively.
4. The energy absorption capacities of HL‐PS and HL‐IS exceed the sum of their individual components by 10.50% and 30.16%, respectively, confirming synergistic interaction effects.

These findings confirm the effectiveness of the design in achieving both high mechanical performance and structural tunability. This study establishes a versatile design pathway for multifunctional lattice metamaterials, with promising applications in adaptive mechanics, acoustic control, intelligent infrastructure, and biomedical engineering.

## 6 Experimental Section

### Specimen Fabrication

The structures designed in this study were manufactured using the PolyJet technique on a 3D printing machine, utilizing a resin‐based Polycarbonate composite polymer (PC+). The machine was notable for its precision, offering a horizontal build layer thickness of 25 µm and a build resolution of 0.03 mm, which were critical for achieving the desired fidelity in the produced structures. Two types of hybrid lattice structures, HL‐PS and HL‐IS, were prepared, with a minimum channel size of 800 µm, ensuring the egress of excess material through the apertures. Notably, due to boundary effects, the number of unit cells in a sample may influence its mechanical response. Research indicates that a 4 × 4 × 4 lattice arrangement sufficiently characterizes mechanical properties, balancing cost and the resemblance to an infinite array.^[^ ^41^ ^]^ The lattice samples studied had dimensions of 60 mm × 60 mm × 60 mm. They closely matched the CAD models, and despite some residual material slightly increasing their weight, the quality variance was kept within 5%. Actual mass measurements and dimensional verifications were provided in Section 5 of the Supporting Information. The fabrication of these hybrid metamaterials began with the construction of customizable SC‐plates and TPMS lattice metamaterials, followed by recording the topological features in the standard.stl file format for rapid prototyping processes. All completed procedures were executed using MATLAB software. To ascertain the material properties of the PC+ substrate employed in this research, tensile tests on dog‐bone specimens were performed according to ASTM D638 standards^[^ ^45^ ^]^ (Section 4, Figure S3, Supporting Information).

### Quasi‐Static Compressions

Quasi‐static compression tests were conducted to analyze the crushing behavior of the proposed samples. All compression tests were conducted on the INSTRON 3367 electro‐universal testing machine with a loading capacity of 30 kN. Before testing, the specimens were placed flat on the support platform, ensuring the centers of the specimens and platform aligned without the need for special fixtures. The tests were performed under displacement control with a strain rate set at 1 × 10^−3^ s^−1^. A rigid block on the loading end compressed the specimens at a constant speed of 3.6 mm min^−1^.^[^ ^64^ ^]^ Throughout the quasi‐static compression process, the deformation patterns of the specimens were photographed. To evaluate the energy absorption characteristics of these structures, the resilience indicators mean plateau stress (*σ*_m_), energy absorption (*J*_E_), specific energy absorption (*SEA*), crush stress efficiency (*ξ*), and smoothness coefficient (*ω*) were calculated using Equations ((6), (7), (8), (9)). The compression test results for the same sample include the mean as well as the standard deviation error. *J*_E_ represents the effective energy absorption per unit volume before densification strain *ε*_d_, corresponding to the area under the stress *σ* versus strain *ε* curve, calculated using Equation 6.

(6) $$J_{E}=\int_{0}^{\varepsilon_{d}}\sigma (\varepsilon )d\varepsilon$$

*SEA* was another crucial indicator to evaluate the energy absorption capacity of a structure. *SEA* was defined as the ration of the total energy absorbed per unit mass to the equivalent density of the lattice.

(7) $$SEA=\frac{J_{E}}{\rho *\rho }$$

where *ρ* was the relative density of the lattice, ρ* was the matrix material density.

The mean plateau stress (*σ*_m_) was a critical indicator to evaluate the compressive strength of structures, which was expressed as the energy absorption *J*_E_ to densification strain *ε*_d_ (*J*_E_/*ε*_d_). Crush stress efficiency (*ξ*), which was defined as the ratio of the mean plateau stress *σ*_m_ to the initial ultimate stress *σ*_u_. The higher the value of *ξ*, the better the load consistency, was given by Equation 8.

(8) $$\xi =\frac{\sigma_{m}}{\sigma_{u}}$$

The smoothness coefficient ω was introduced to quantitatively evaluate the fluctuation degree of the stress versus strain curve, reflecting the structural stability under load conditions. It was defined as:

(9) $$\omega =\frac{\sigma_{m}}{\sigma_{max}-\sigma_{min}}$$

where *σ*_max_ and *σ*_min_ mean the maximum and minimum stresses in the yield stage, respectively.

### Finite Element Analysis

Finite element analysis (FEA) was adopted to compare the crushing behavior of the two types of TPMS‐plate lattices with their original lattice counterparts. This comparison aims to further explore their mechanical advantages and potential energy absorption mechanisms. The material property of the structure was modeled as ideal elastoplastic solids, with the simulated material characteristics defined by the true stress‐strain curve (Figure S3, Supporting Information). The material properties specified include a Young's modulus of 1.94 GPa, yield strength of 27.40 MPa, density of 1.31 g cm^−3^, and a Poisson's ratio of 0.3. The models employ C3D4 4‐nodes tetrahedral elements with a maximum element size of 0.6 mm, achieving improved accuracy in both stress history and densification strain.^[^ ^65^ ^]^ Developed in MATLAB and saved as STL files, the models were imported into HYPERMESH for grid redistribution (Section 2, Supporting Information), optimizing the balance between computational efficiency and data precision. The models were further developed in ABAQUS/Explicit to establish the quasi‐static compression framework, with the lower plate fully fixed as a rigid body. To enhance computational efficiency, the upper rigid plate was constrained vertically, with a constant compression speed of 5 m/s. A general contact with a friction coefficient of 0.2 was defined. To verify the reliability and accuracy of the finite element model, this study evaluated the mechanical response of hybrid lattices based on experimental data (Section 6, Supporting Information).

## Conflict of Interest

The authors declare no conflict of interest.

## Supporting information



Supporting Information

Supplemental Video 1

Supplemental Video 2

## Data Availability

The data that support the findings of this study are available from the corresponding author upon reasonable request.
