# Supplementary material for: Breaking Stiffness‐Tunability Trade‐offs in Metamaterials: a Minimal Surface Guided Hybrid Lattice Strategy
Source: Adv Sci (Weinh). 2025 Aug 11;12(39):e10586. doi: 10.1002/advs.202510586 (PMC12533393; doi:10.1002/advs.202510586)
Supplement: Supplementary file 1 — Supporting Information [file ADVS-12-e10586-s001.docx]

**Supplementary Information**

**Breaking Stiffness-Tunability Trade-offs in Metamaterials: A Minimal Surface Guided Hybrid Lattice Strategy**

Min Zhang ^a^, Kang Gao ^a*^, Jinlong Liu ^a^, Zhiqiang Zou ^a^, Jie Yang ^c^, Ma Qian ^c^, Wei Zhai ^d^, Zhangming Wu ^b^

^a^ Key Laboratory of Concrete and Prestressed Concrete Structures of the Ministry of Education, School of Civil Engineering, Southeast University, Nanjing, China

^b^ School of Engineering, Cardiff University, The Parade, Cardiff CF24 3AA, UK

^c^ School of Engineering, RMIT University, Melbourne, VIC 3000, Australia

^d^ Department of Mechanical Engineering, National University of Singapore, Singapore, Singapore

# Section S1. Examples of original metamaterials and hybrid metamaterials under different parameter controls

For ease of description and documentation, different types of specimens are named specifically. For instance, “HL-PS” specimen indicate a combination of Primitive-type TPMS lattice and SC-plate lattice, while “HL-IS” specimen represent a combination of IWP-type TPMS lattice and SC-plate lattice. The various types of unit cells controlled by different parameters can be found (Table S1).

**Table S1.** Details of different types of unit cells

| Sample | Parameter | | Relative density | Characteristics | Unit cell |
| --- | --- | --- | --- | --- | --- |
| Primitive | | *T*_1_ = 0.2; *t*_1_ = -0.5 | 0.11 | Primitive | 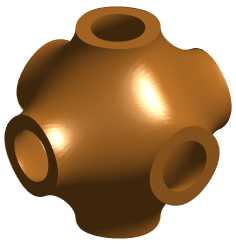 |
| IWP | | *T*_2_ = 0.2; *t*_2_ = -1 | 0.14 | IWP | 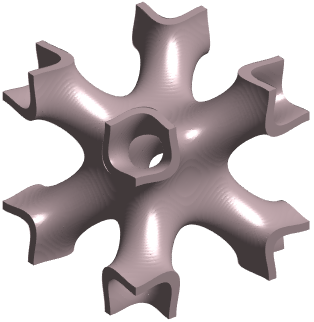 |
| Plate | | *T*_3_ = 1; *t*_3_ = 0.5 | 0.17 | SC-Plate | 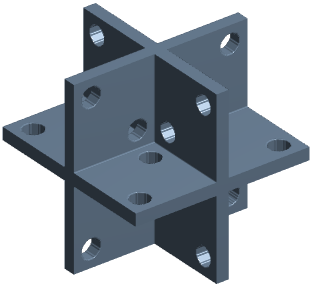 |
| HL-PS | | *T*_1_ = 0.2; *t*_1_ = 0.3;  *T*_3_ = 0.8; *t*_3_ = 0.4 | 0.27 | shell-like Primitive lattice+SC-plate | 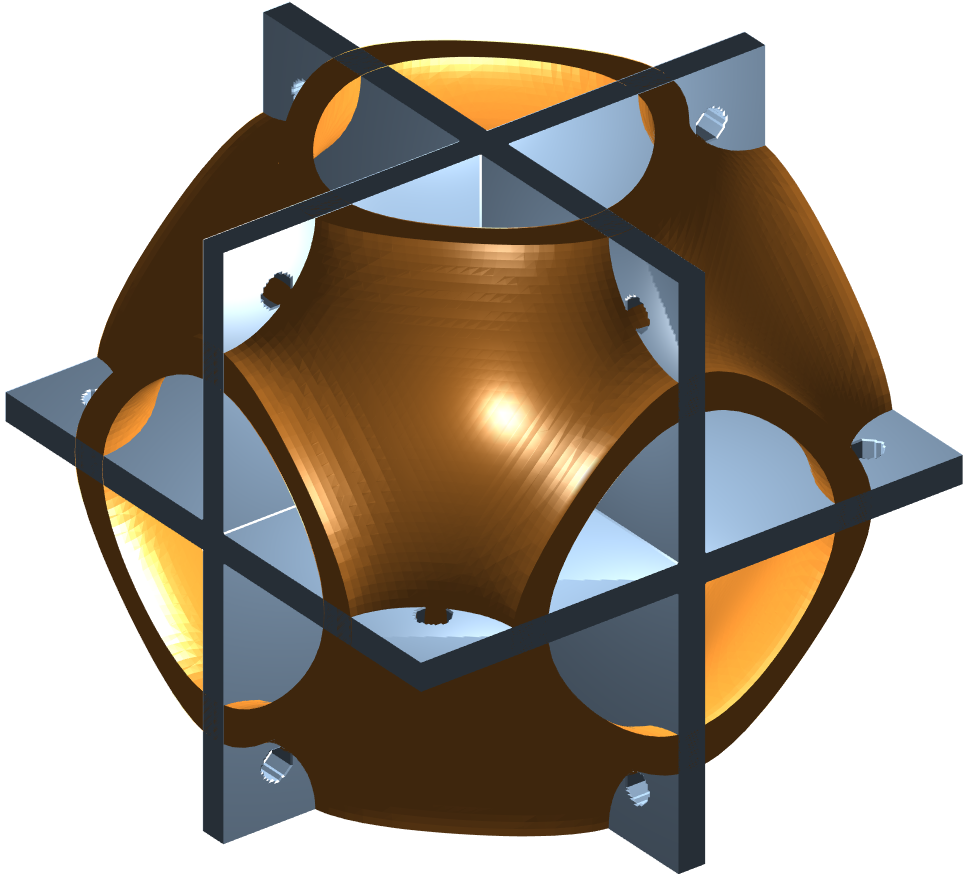 |
| HL-PS | | *T*_1_ = 0.4; *t*_1_ = -1;  *T*_3_ = 1.4; *t*_3_ = 0.4 | 0.36 | truss-like Primitive lattice+SC-plate | 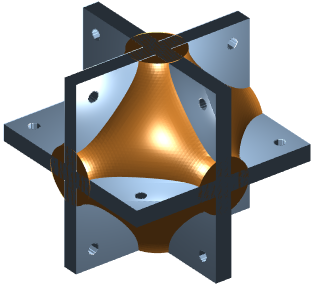 |
| HL-PS | | *T*_1_ = 0.2; *t*_1_ = -0.6;  *T*_3_ = 1.0; *t*_3_ = 0.6 | 0.26 | hollow truss-like Primitive lattice+SC-plate | 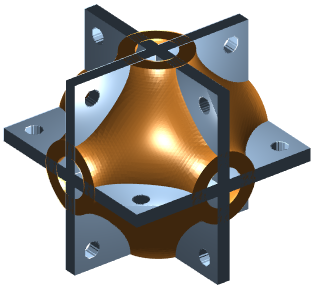 |
| HL-IS | | *T*_2_ = 0.2; *t*_2_ = 0.8;  *T*_3_ = 0.8; *t*_3_ = 0.8 | 0.25 | shell-like IWP lattice+SC-plate | 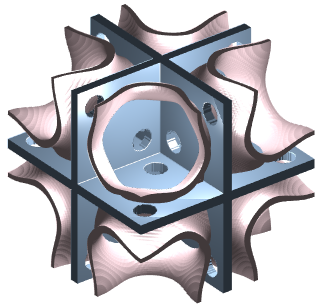 |
| HL-IS | | *T*_2_ = 0.4; *t*_2_ = -1.2  *T*_3_ = 1.2; *t*_3_ = 0.6; | 0.50 | truss-like IWP lattice+SC-plate | 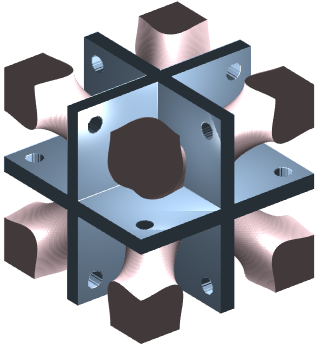 |
| HL-IS | | *T*_2_ = 0.2; *t*_2_ = -0.8  *T*_3_ = 1.2; *t*_3_ = 0.6; | 0.35 | hollow truss-like IWP lattice +SC-pate | 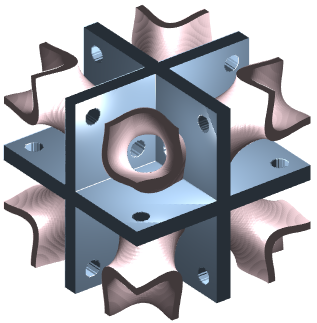 |

# Section S2. Practical implementation of design strategies for hybrid lattices

To verify the effectiveness of the design strategy, a variety of software tools were employed for practical implementation. As illustrated in Figure S1, we initially used MATLAB to generate candidate TPMS surfaces based on level set functions, and to modify nodes, lines and surfaces to create plate cells. Hybridization was then applied by combining the dislocations to generate a ‘stl.’ document. Critically, this file was imported into HYPERMESH where the geometric models of the plate lattice and TPMS lattice underwent Boolean fusion operations. This process created a single, watertight, and topologically continuous solid model – eliminating any artificial interfaces between the two lattice types. (Pre-fusion components were distinctly colored; post-fusion model exhibits uniform coloration confirming seamless integration), followed by a redefinition of the mesh to ensure high quality, considering both computational resources and result accuracy. Finally, numerical simulations were conducted using the commercial software ABAQUS.

| 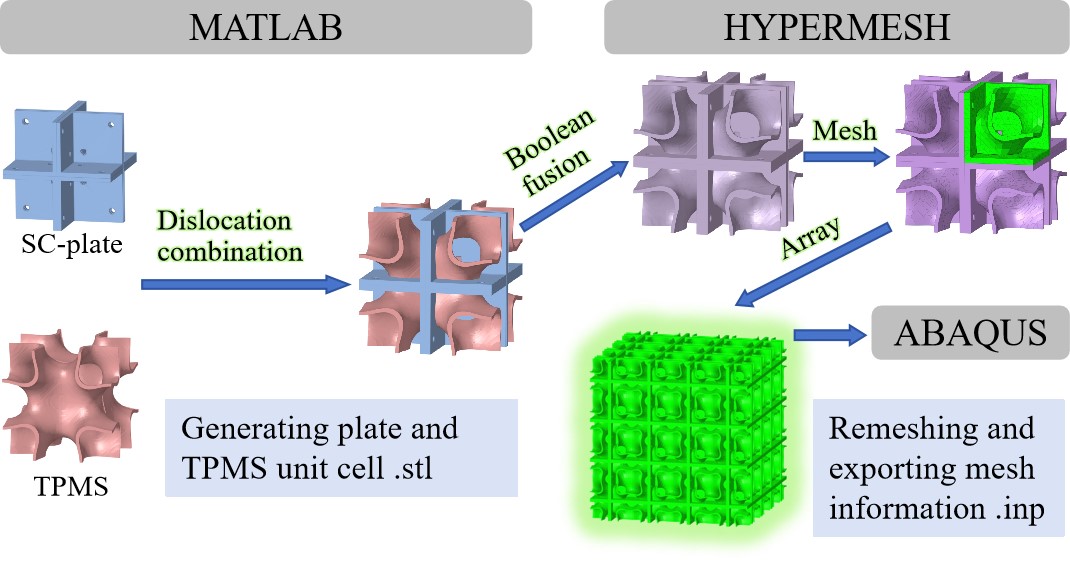 |
| --- |
| **Figure S1.** Practical implementation flowchart of the hybrid lattices |

## Section S3. Voxel-based homogenization method

Homogenization methods are typically employed to solve general boundary value problems characterized by periodic boundary conditions ^[1,2]^. In the study of three-dimensional periodic lattice structures, the homogenization process relies on finite element methods and energy homogenization theory. Specifically, the periodic boundary domain *Ω* is discretized into finite elements *e*. For each element, the components of the homogeneous material tensor in the microstructures of the periodic unit cells, indexed by *i*, *j*, *k*, *l* ∈ {1, 2, 3}, can be approximated as follows:

| $C_{ijkl}^{H}=\frac{1}{\vert\Omega\vert}\int_{e\in\Omega} \left(\overline{\varepsilon_{e}^{(ij)}}-\varepsilon_{e}^{(ij)}(u)\right)^{T}C_{e}^{b}\left(\overline{\varepsilon_{e}^{(kl)}}-\varepsilon_{e}^{(kl)}(u)\right)\text{d}\Omega$ | (S1) |
| --- | --- |

Where is the stiffness tensor of the base material in the microstructure. The volume of the domain, denoted as *|Ω|*, is defined by the boundaries of the unit cell. $\overline{\varepsilon}$ represents the applied strain fields at the macroscopic scale, while ***ε***(***u***) denotes the locally varying strain fields. These can be derived through the principle of virtual work.

| $\int_{e\in\Omega} C_{e}^{b}\varepsilon_{e}^{(ij)}(v)\varepsilon_{e}^{(kl)}(u)\text{d}\Omega=\int_{e\in\Omega} C_{e}^{b}\varepsilon_{e}^{(ij)}(v)\overline{\varepsilon_{e}^{(kl)}}\text{ d}\Omega$ | (S2) |
| --- | --- |

In the formula, *v* represents the virtual displacement field, ***u*** denotes the local displacement fields under the specified macroscopic strain fields. For each element *e* within the domain *Ω*, the global stiffness matrix **K** and the force vector ***f*** can be derived using the following equations:

| *$K_{e}=\sum_{e\in\Omega} B_{e}^{T}C^{b}B_{e} \text{d}\Omega$* | (S3) |
| --- | --- |
| *$f_{e}=\sum_{e\in\Omega} B_{e}^{T}C^{b}\overline{\varepsilon}d\Omega$* | (S4) |

**B***_e_* is the strain–displacement matrix for element *e*, and ***ε***_e_(***u***)= **B**_e_***u***. The strain fields at the macroscopic scale can be decomposed into six independent vectors.

| $\begin{matrix} \bar{\varepsilon}_{1}=\{1,0,0,0,0,0\},\bar{\varepsilon}_{2}=\{0,1,0,0,0,0\}, \\ \bar{\varepsilon}_{3}=\{0,0,1,0,0,0\},\bar{\varepsilon}_{4}=\{0,0,0,1,0,0\}, \\ \bar{\varepsilon}_{5}=\{0,0,0,0,1,0\},\bar{\varepsilon}_{6}=\{0,0,0,0,0,1\}. \end{matrix}$ | (S5) |
| --- | --- |

The local microscale displacement field u can be calculated by solving the linear equation system **K*u***=***f***. In the context of linear elasticity, the local strain fields ***ε***(***u***) for each element should be linearly related to the macroscopic strain field **$\overline{\varepsilon}$**:

| $A_{e}=\bar{\varepsilon}-\varepsilon_{e}\left(u_{e}\right)=\bar{\varepsilon}-B_{e}u_{e}$ | (S6) |
| --- | --- |

According to (, the effective elastic tensor **C***^H^* of the periodic microstructured material can be provided by an integration step:

| $C^{H}=\frac{1}{\vert\Omega\vert}\sum_{\varepsilon\in\Omega} \int A_{e}^{T}C^{b}A_{e}\text{d}\Omega=\frac{1}{\vert\Omega\vert}\sum_{\varepsilon\in\Omega} \int\left(\overline{\varepsilon}-\varepsilon_{e}(u_{e})\right)^{T}C^{b}\left(\overline{\varepsilon}-\varepsilon_{e}(u_{e})\right)\text{d}\Omega$ | (S7) |
| --- | --- |

The calculations of the homogenized elastic tensor for three-dimensional cellular materials were conducted in MATLAB®. The input model is a 3D voxel model derived from a discretized lattice structure, with isotropic material properties maintained in each element. A regular grid of 64×64×64 with eight-node hexahedral elements was utilized, a resolution that is considered optimal for balancing computational accuracy and processing speed. Subsequently, isotropic materials were used as the base in the homogenization computation process, characterized by a Young’s modulus of 1 MPa and a Poisson's ratio of 0.3. In this study, the original lattices and hybrid lattice structures approximate an orthotropic isotropic material. For metamaterials with triaxial symmetry, owing to the congruent geometry along the axis directions, these are consistently modeled as orthotropic materials ^[3]^. The linear elastic stress-strain relationship is characterized as follows:

| $\left\{\begin{matrix} \sigma_{11} \\ \sigma_{22} \\ \sigma_{33} \\ \sigma_{23} \\ \sigma_{31} \\ \sigma_{12} \end{matrix}\right\}=\left[\begin{matrix} C_{11} & C_{12} & C_{12} & 0 & 0 & 0 \\ C_{12} & C_{11} & C_{12} & 0 & 0 & 0 \\ C_{12} & C_{12} & C_{11} & 0 & 0 & 0 \\ 0 & 0 & 0 & C_{44} & 0 & 0 \\ 0 & 0 & 0 & 0 & C_{44} & 0 \\ 0 & 0 & 0 & 0 & 0 & C_{44} \end{matrix}\right]\left\{\begin{matrix} \varepsilon_{11} \\ \varepsilon_{22} \\ \varepsilon_{33} \\ \varepsilon_{23} \\ \varepsilon_{31} \\ \varepsilon_{12} \end{matrix}\right\}$ | (S8) |
| --- | --- |

The stiffness matrix obtained from numerical homogenization calculations serves to evaluate the elastic properties of materials. For three-dimensional orthotropic materials, Young’s modulus (*E*), and the Zener anisotropy ratio (*Z*) for evaluating the anisotropy can be computed from the matrix as illustrated. Figure S2 demonstrates the process of utilizing a homogenized constitutive model to predict the elastic characteristics of lattice structures in various orientations.

| $E=\left(C_{11}^{2}+C_{12}C_{11}-2C_{12}^{2}\right)/\left(C_{11}+C_{12}\right)$ | (S9) |
| --- | --- |
| $Z=2C_{44}/\left(C_{11}-C_{12}\right)$ | (S10) |

| 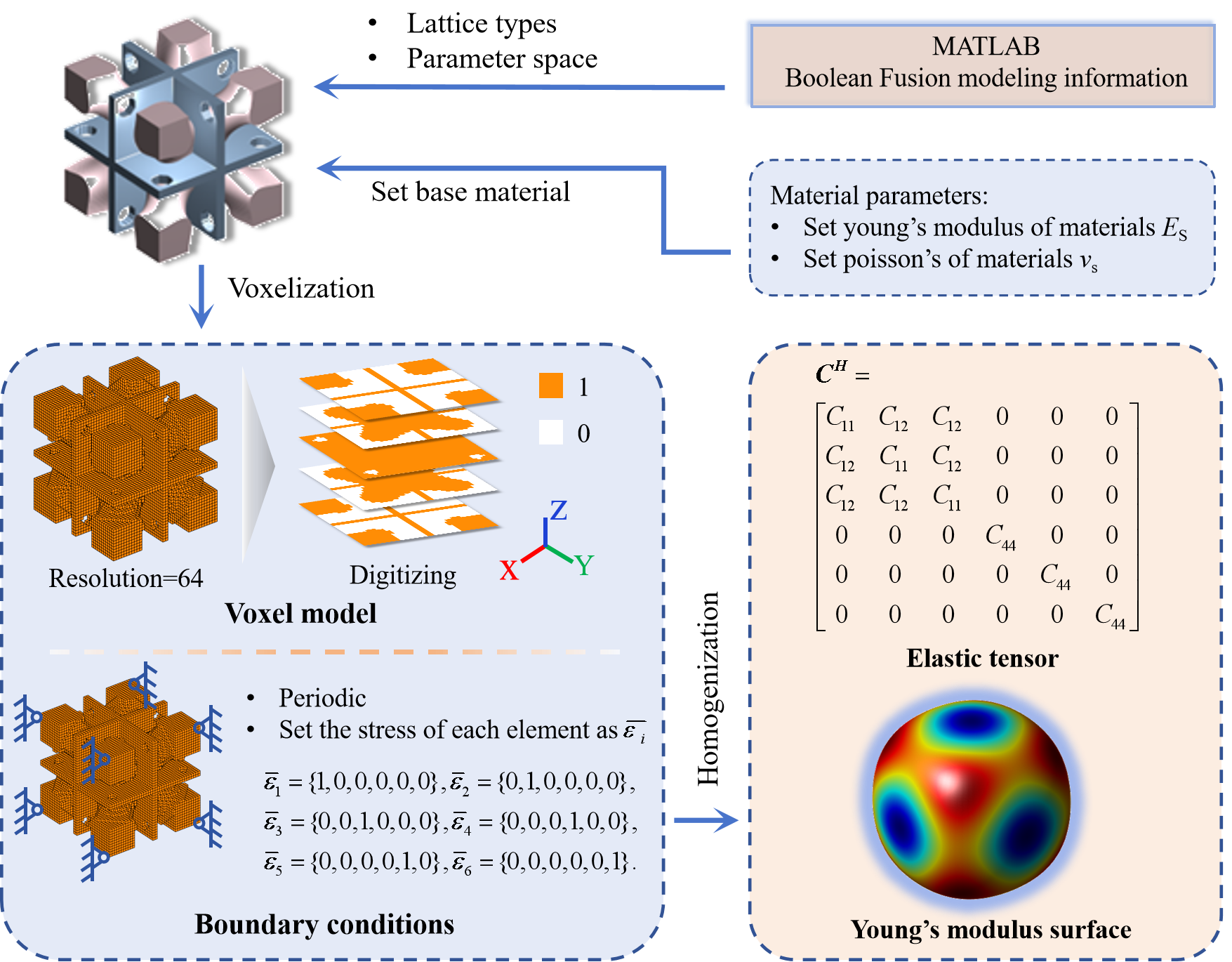 |
| --- |
| **Figure S2.** Homogenization method calculation steps |

# Section S4. Materials properties of Polycarbonate (PC) composite polymers (PC+) obtained through tensile tests

The PolyJet 3D printing technology used in this study involves layer-by-layer deposition of micro-droplets, which are instantly cured by ultraviolet (UV) light, thus the mechanical performance of the printed structures is influenced by the orientation of the build ^[4]^. The build orientation was maintained parallel to the load direction throughout the experiments. The characteristic tensile stress-strain curve for the utilized printing substrate is shown in Figure S3(a), with a material density of approximately 1.31 g/cm^3^. Given the sample size and fabrication precision, the lattice structures created via PolyJet 3D printing were effectively implemented.

| 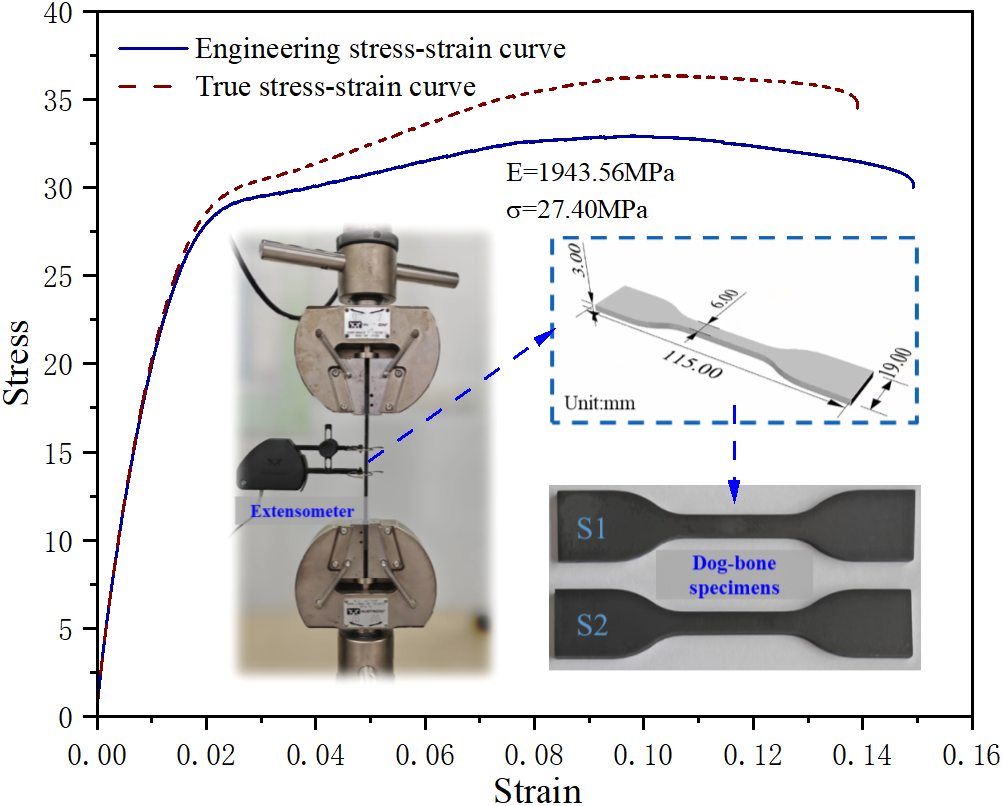 | 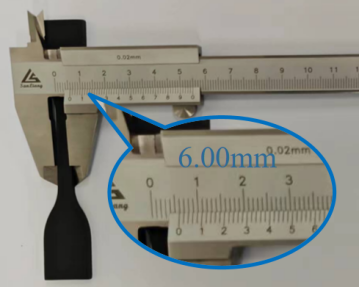 |
| --- | --- |
|  | 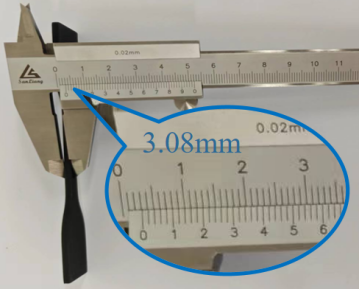 |
| (a) | (b) |
| **Figure S3.** Details of the dog bone tensile test: (a) tensile stress-strain curves for PC+ materials, (b) dimensions (in mm) of the dog-bone specimens, according to the ASTM standard. | |

# Section S5. Fabricated lattice models and mass comparison

**
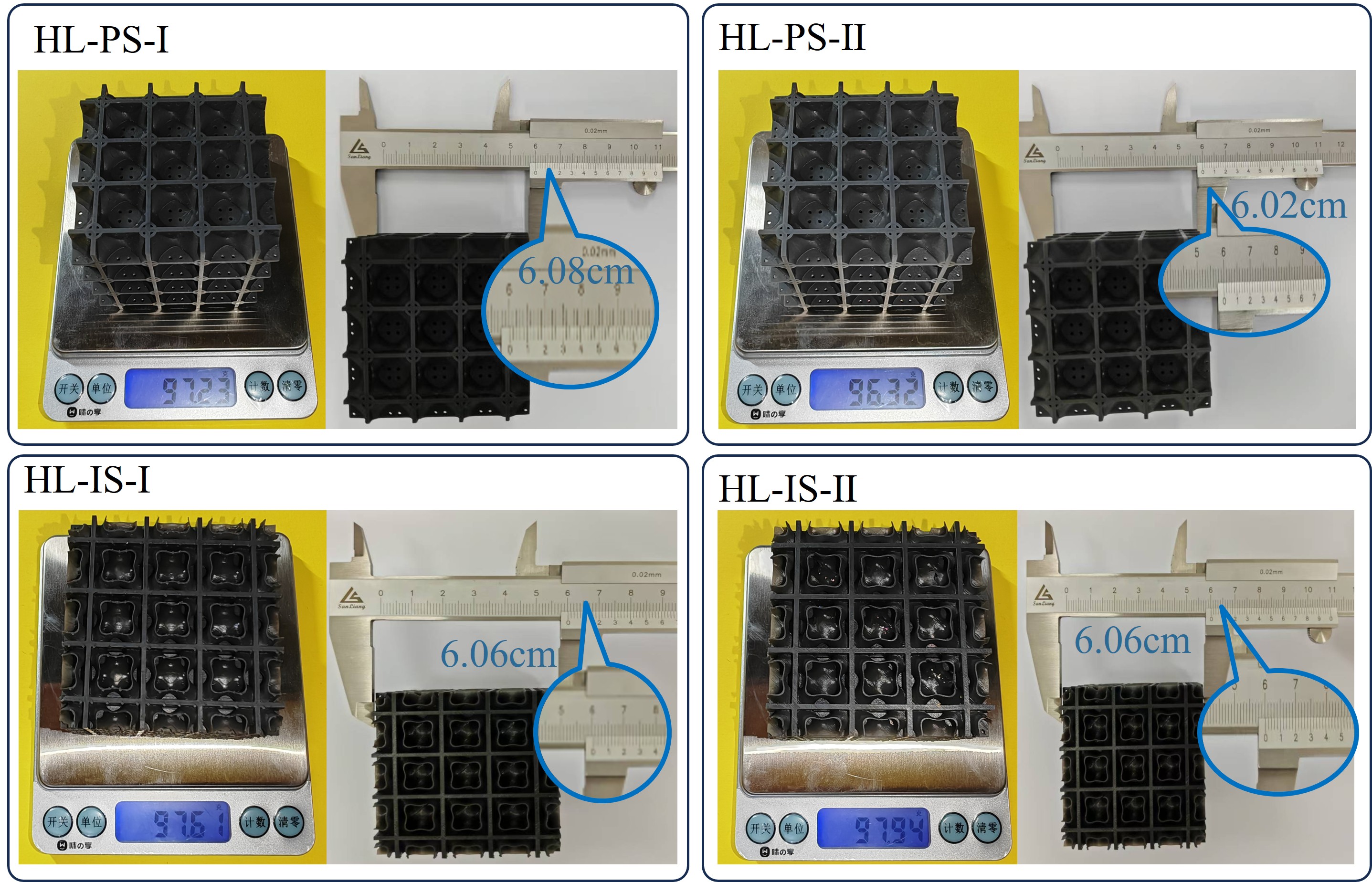
**

**Figure S4.** Fabricated cell models and mass comparison: printed cell models with TPU

**Table S2.** Mass of printed sample

| Sample | Relative density | | Theoretical mass (g) | Actual mass (g) | Error |
| --- | --- | --- | --- | --- | --- |
| HL-PS-Ⅰ | | 0.33 | 93.38 | 97.23 | 4.12% |
| HL-PS-Ⅱ | | 0.33 | 93.38 | 96.32 | 3.15% |
| HL-IS-Ⅰ | | 0.34 | 96.21 | 97.61 | 1.46% |
| HL-IS-Ⅱ | | 0.34 | 96.21 | 97.94 | 1.80% |

# Section S6. Validation of the finite element models

To verify the reliability and accuracy of the finite element model, this study evaluated the mechanical response of the HL-IS specimens based on experimental data, as shown in Figure S5. Good agreement was achieved between the experimental and numerical results concerning the peak, fluctuating, and plateau force. Furthermore, a comparison of deformation patterns under varying strain conditions revealed that the occurrence of folds in the plate corresponded well with the simulation predictions. While the HL-IS lattice exhibited distinct plateau behavior in the finite element simulation, the experimental stress-strain curves showed a 18.91% reduction in stress during the initial plateau phase. This phenomenon is primarily attributed to debonding of the layer interface and surface defects, which are common in 3D printed samples due to the layer-stacked process of resin materials ^[5]^. Finite element analysis was used to explore the deformation patterns of both the original and hybrid lattices. The overall deformation pattern (layer-by-layer crushing) and key damage feature (localized folding) showed good agreement between the finite element results and experimental data. Although there are slight differences in the localized folding direction (outward or inward), the errors in key energy dissipation indicators were within 10% (Table S3).

| \| 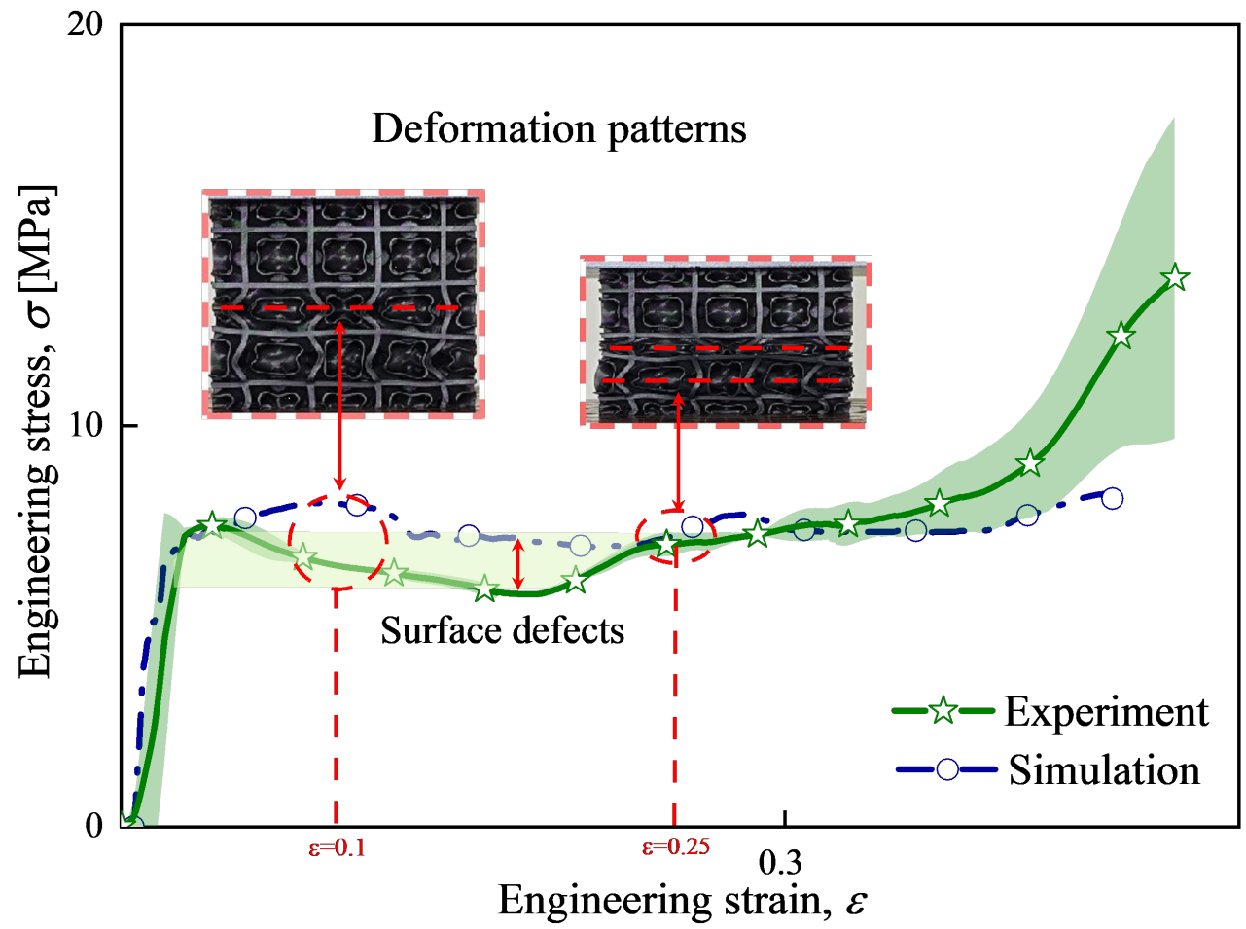 \| **Table S3.** The comparison of experimental and simulated key mechanical indices of HL-IS specimens.   \| **Indicators** \| **Exp.** \| **FEA** \| **Error** \| \| --- \| --- \| --- \| --- \| \| *σ*_u_ (MPa) \| 7.56 \| 8.08 \| 6.64% \| \| *σ*_m_(MPa) \| 7.11 \| 7.24 \| 1.80% \| \| *J*_E_(×10^3^kJ/m^3^) \| 2.44 \| 2.64 \| 7.58% \| \| *SEA*(J/g) \| 6.24 \| 6.75 \| 7.55% \| \| \| --- \| --- \| --- \| --- \| --- \| --- \| --- \| --- \| --- \| --- \| --- \| --- \| --- \| --- \| --- \| --- \| --- \| --- \| --- \| --- \| --- \| --- \| \| **Figure S5.** The comparison of stress-strain curves between experiments and simulation of HL-IS specimens. \| \| |
| --- | --- | --- | --- | --- | --- | --- | --- | --- | --- | --- | --- | --- | --- | --- | --- | --- | --- | --- | --- | --- | --- | --- | --- | --- |
| 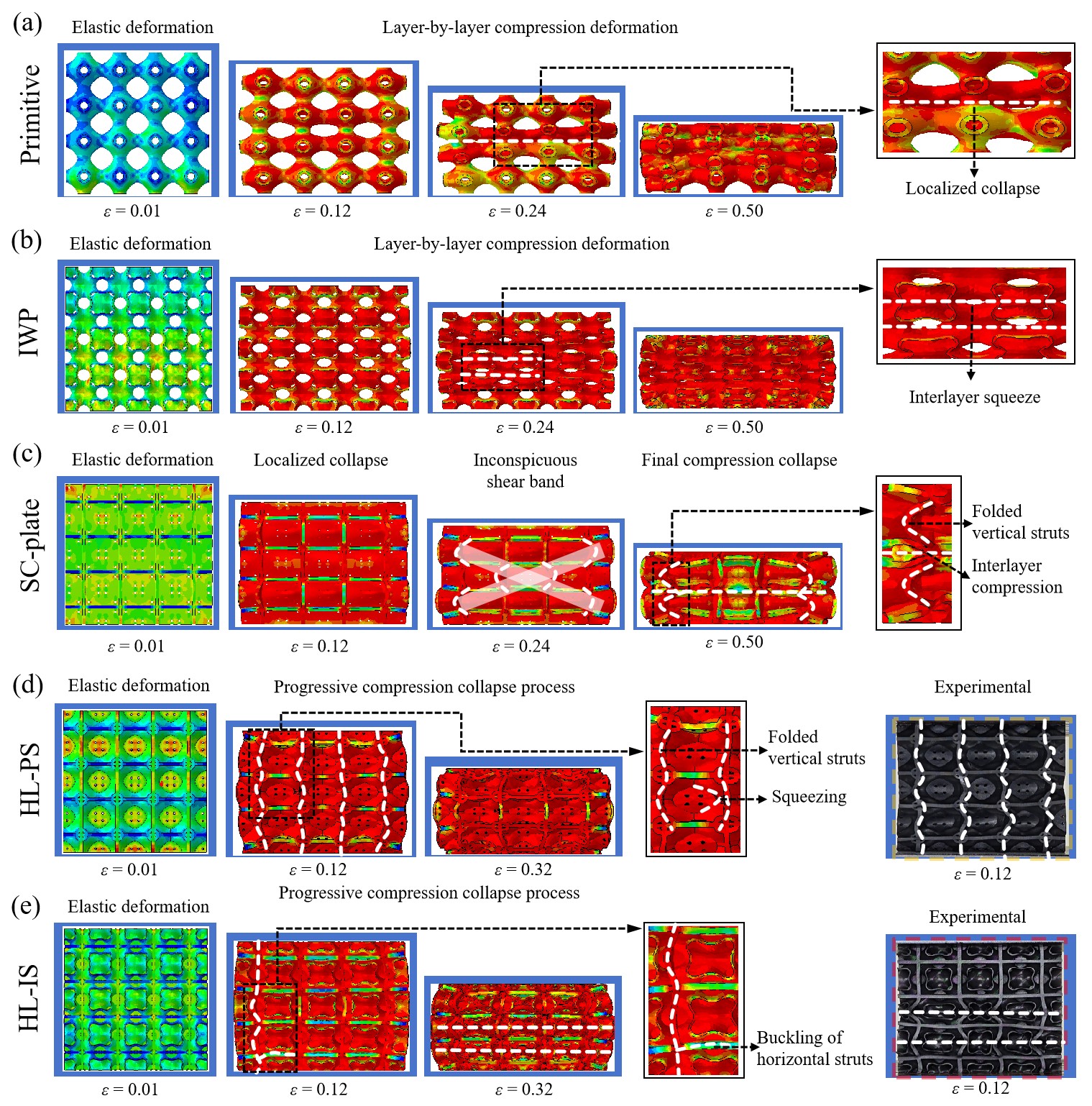 |
| **Figure S6.** Simulated deformation pattern results for the original lattices and hybrid lattices |

# Section S7. Supplementary videos

**Video S1.** *Quasi-static compression response of the sample HL-PS*

**Video S2.** *Quasi-static compression response of the sample HL-IS*

**References**

[1] Y. Xu, H. Pan, R. Wang, Q. Du, L. Lu, *Additive Manufacturing* **2023**, *77*, 103779.

[2] P. Liu, B. Sun, J. Liu, L. Lu, *Additive Manufacturing* **2022**, *60*, 103258.

[3] R. Chen, W. Zhang, Y. Jia, S. Wang, B. Cao, C. Li, J. Du, S. Yu, J. Wei, *Materials & Design* **2024**, *244*, 113107.

[4] S. Wang, M. Zhang, W. Pei, F. Yu, Y. Jiang, *Composite Structures* **2022**, *299*, 116149.

[5] L. Chen, J. Zhang, B. Du, H. Zhou, H. Liu, Y. Guo, W. Li, D. Fang, *Thin-Walled Structures* **2018**, *127*, 333.
